# Early and extensive alterations of glial connexins, distal oligodendrogliopathy type demyelination, and nodal/paranodal pathology are characteristic of multiple system atrophy

**DOI:** 10.1111/bpa.13131

**Published:** 2022-11-11

**Authors:** Yuji Nishimura, Katsuhisa Masaki, Dai Matsuse, Hiroo Yamaguchi, Tatsunori Tanaka, Eriko Matsuo, Shotaro Hayashida, Mitsuru Watanabe, Takuya Matsushita, Shoko Sadashima, Naokazu Sasagasako, Ryo Yamasaki, Noriko Isobe, Toru Iwaki, Jun‐ichi Kira

**Affiliations:** ^1^ Department of Neurology, Neurological Institute, Graduate School of Medical Sciences Kyushu University Fukuoka Japan; ^2^ Sumitomo Pharma Osaka Japan; ^3^ Department of Neuropathology, Neurological Institute, Graduate School of Medical Sciences Kyushu University Fukuoka Japan; ^4^ Department of Neurology, Neuro‐Muscular Center National Omuta Hospital Fukuoka Japan; ^5^ Translational Neuroscience Center, Graduate School of Medicine, and School of Pharmacy at Fukuoka International University of Health and Welfare Ookawa Japan; ^6^ Department of Neurology, Brain and Nerve Center Fukuoka Central Hospital Fukuoka Japan

**Keywords:** connexin, distal oligodendrogliopathy, gap junction, multiple system atrophy, synuclein

## Abstract

The pathological hallmark of multiple system atrophy (MSA) is aberrant accumulation of phosphorylated α‐synuclein in oligodendrocytes, forming glial cytoplasmic inclusions (GCIs). Extensive demyelination occurs particularly in the olivopontocerebellar and striatonigral pathways, but its precise mechanism remains elusive. Glial connexins (Cxs), which form gap junction channels between astrocytes and oligodendrocytes, play critical roles in myelin maintenance, and have not been studied in MSA. Therefore, we immunohistochemically investigated glial Cx changes in the cerebellar afferent fibers in 15 autopsied patients with MSA. We classified demyelinating lesions into three stages based on Klüver–Barrera staining: early (Stage I), intermediate (Stage II), and late (Stage III) stages showing subtle, moderate, and severe myelin reduction, respectively. Myelin‐associated glycoprotein, but not myelin oligodendrocyte glycoprotein, was preferentially decreased in Stage I, suggesting distal oligodendrogliopathy type demyelination. Accumulation of phosphorylated α‐synuclein in oligodendrocytes was frequently seen in Stage I but less frequently observed in Stages II and III. Tubulin polymerization‐promoting protein (TPPP/p25α)‐positive oligodendrocytes were preserved in Stage I but successively decreased in Stages II and III. Even at Stage I, Cx32 was nearly absent from myelin, despite the relative preservation of other nodal proteins, such as neurofascin, claudin‐11/oligodendrocyte‐specific protein, and contactin‐associated protein 1, which successively decreased in the later stages. Cx32 was re‐distributed in the oligodendrocyte cytoplasm and co‐localized with GCIs. Cx47 gradually decreased at the oligodendrocyte surface in a stage‐dependent manner but was not co‐localized with GCIs. Astrocytic Cx43 was down‐regulated in Stage I but up‐regulated in Stages II and III, reflecting astrogliosis. Cx43/Cx47 gap junctions significantly decreased from Stage I to III. Activated microglia/macrophages and T cells infiltrated in Stage I rather than Stages II and III. Therefore, early and extensive alterations of glial Cxs, particularly Cx32 loss, occur in MSA and may accelerate distal oligodendrogliopathy type demyelination and nodal/paranodal dysfunction through disruption of inter‐glial communication.

## Graphical abstract

Early and extensive alterations of glial Cxs, particularly loss of membranous Cx32 in oligodendrocytic somata and myelin and Cx32 re‐distribution to the oligodendrocytic cytoplasm in MSA may accelerate distal oligodendrogliopathy type demyelination and nodal/paranodal dysfunction through disruption of inter‐glial communication.

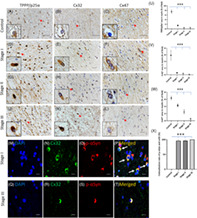

## 1 INTRODUCTION

Multiple system atrophy (MSA) is a rare adult‐onset and lethal neurodegenerative disorder clinically characterized by rapidly progressing parkinsonism, cerebellar ataxia, autonomic dysfunction, and corticospinal tract impairment. The pathological hallmark of MSA is abnormal accumulation of phosphorylated α‐synuclein (p‐αSyn) as glial cytoplasmic inclusions (GCIs) in oligodendrocytes [1], which may represent a primary pathogenic event [2]. Other core features include loss of neurons and myelinated fibers, extensive astrogliosis, and widespread infiltration of activated microglia and macrophages together with CD4‐ or CD8‐positive T cells [3] in multiple regions of the central nervous system (CNS), phenomena that are particularly prominent in the olivopontocerebellar and striatonigral systems [4]. The extensive demyelination pathologically observed in these areas is associated with reductions of up to approximately 50% in myelin constituents including sphingomyelin, sulfatide, and galactoceramide [5]. Various mechanisms of demyelination have been proposed, including abnormal metabolism of myelin lipids [6], mitochondrial abnormalities [7], autophagy dysfunction [8], and re‐localization of myelin proteins such as tubulin polymerization‐promoting protein (TPPP/p25α) to oligodendrocyte somata [9]. However, the precise mechanism of demyelination, including the regional vulnerability of the white matter, remains to be elucidated.

Glial connexins (Cxs) form homotypic gap junctions (GJs) between astrocytes or oligodendrocytes or heterotypic GJs between astrocytes and oligodendrocytes [10]. GJs appose two cells and form channels for direct intercellular communication, through which intracellular second messengers, such as calcium ions and other small molecules, are exchanged [11]. In human demyelinating disorders, we and others have reported the extensive loss of glial Cx43, Cx32, and Cx47 in active demyelinating lesions in cases of multiple sclerosis (MS), neuromyelitis optica spectrum disorder (NMOSD), and Baló's concentric sclerosis, an extremely rare fulminant demyelinating disease [12, 13, 14]. Experimentally, astrocytic Cx43 and Cx30, oligodendrocytic Cx32 and Cx47, and astrocytic Cx43 and oligodendrocytic Cx32 double knockout (dKO) mice showed thin or absent myelin sheaths, vacuolation, and enlarged periaxonal collars, leading to oligodendrocyte death and axonal loss [15, 16, 17]. These observations indicate the critical roles of astrocytic and oligodendrocytic Cxs in the maintenance and loss of CNS myelin.

Recent reports have indicated that uptake of αSyn secreted from neurons by oligodendrocytes is facilitated through direct protein–protein interaction between oligomeric αSyn and Cx32 [18]. Involvement of glial Cxs, such as Cx32, is thus suggested in the pathomechanism of MSA; however, to the best of our knowledge, alterations of glial Cxs have not been reported in this condition, particularly in the context of demyelination. Therefore, in the present study, we aimed to characterize Cx changes from early to late demyelinating lesions in MSA by systematic investigation of the expression of glial Cxs relative to other oligodendrocytic/myelin and astrocytic proteins. Furthermore, because we previously reported that astroglial Cx43 was down‐regulated by interferon‐γ produced by Th1 cells via activation of microglia [19], the degree of immunocyte infiltration, including T cells, B cells, and macrophages/microglia, was also measured to identify any association between glial Cx expression and inflammatory cell infiltration.

## 2 MATERIALS AND METHODS

### 2.1 Autopsy samples

Immunohistological studies were performed on archival autopsied brain specimens of patients clinically diagnosed and pathologically confirmed to have MSA at National Hospital Organization Omuta Hospital or Kyushu University Hospital using the diagnostic criteria for MSA [20, 21]. We examined 15 MSA specimens. The age at autopsy ranged from 54 to 81 years for MSA patients (10 men and 5 women), and the disease duration ranged from 3 to 15 years (Table 1). The control specimens came from individuals with Becker muscular dystrophy (73 years old), limb‐girdle muscular dystrophy (73 years old), myotonic dystrophy (58 years old), and Duchenne muscular dystrophy (16 years old). Although the mean age at autopsy was younger in controls than in MSA cases (48 versus 67 years old), the difference was not significant (Mann–Whitney *U* test). Moreover, the pathological findings of the pons and cerebellum from the youngest control were similar to those of the other control cases.

**TABLE 1:** Demographic features of autopsied MSA patients

Autopsied cases	Age (yrs)	Sex	Disease duration (yrs)	Brain weight (g)	PMI (hour)	Initial symptom	GCI distribution in olivopontocerebellar circuits	Diagnosis	Pathological stage (olivopontocerebellar circuits)
MSA‐1	81	M	24	1170	2.25	Ataxic gait, dysarthria	+	MSA‐C	III
MSA‐2	64	M	6	1350	11	Ataxic gait, orthostatic hypotension	+	MSA‐C	I
MSA‐3	83	M	6	1206	10	Ataxic gait	+	MSA‐C	II
MSA‐4	78	M	13	1311	9.6	Ataxic gait, dysarthria	+	MSA‐C	III
MSA‐5	70	F	15	1226	1.15	Ataxic gait, dysgraphia	+	MSA‐C	II
MSA‐6	64	F	5	1130	14.8	Ataxic gait, vertigo	+	MSA	I
MSA‐7	85	F	15	1270	5.2	Ataxic gait, dysarthria	+	MSA‐C	III
MSA‐8	69	M	6	1320	34	Parkinsonism	+	MSA‐P	I
MSA‐9	82	F	13	1140	6	Parkinsonism	+	MSA‐P	III
MSA‐10	82	F	18	900	3.5	Ataxic gait	+	MSA‐C	III
MSA‐11	62	M	3	1320	N.D.	Dysuria, orthostatic hypotension	+	MSA‐P	II
MSA‐12	62	M	3	1340	3	Orthostatic hypotension, gait disturbance	+	MSA‐P hypoxia	I
MSA‐13	74	M	10	1220	4.5	Ataxic gait	+	MSA‐C	III
MSA‐14	54	M	4	1470	11	Parkinsonism	+	MSA‐P	I
MSA‐15	59	M	13	1290	3	Ataxic gait	+	MSA‐C	III

Abbreviations: F, female; GCI, glial cytoplasmic inclusion; M, male; MSA, multiple system atrophy; MSA‐C, multiple system atrophy, cerebellar variant; MSA‐P, multiple system atrophy, parkinsonian variant; N.D., not described; PMI, postmortem interval; yrs, years; +, detected.

### 2.2 Tissue preparation and immunohistochemistry

Autopsy specimens were fixed in 10% buffered formalin and processed into paraffin sections. The sections were routinely subjected to hematoxylin and eosin, Klüver–Barrera (KB), and Gallyas silver staining. The primary antibodies used for immunohistochemistry are listed in Table 2. Cx43 and Cx30 were considered astrocytic Cxs, while Cx32 and Cx47 were considered oligodendrocytic Cxs. Purinergic receptor P2RY12 was used as a homeostatic microglia marker and transmembrane protein 119 (TMEM119) was used as an activated microglia marker, as previously reported [22]. CD68 was used as a microglia/macrophage marker [23]. All sections were deparaffinized in xylene and rehydrated through a graded ethanol series. Endogenous peroxidase activity was blocked with 0.3% (v/v) H_2_O_2_/methanol. The sections were then incubated with a primary antibody at 4°C overnight. After rinsing, sections were incubated with enhanced indirect immunoperoxidase reagents using an Envision system (DakoCytomation, Glostrup, Denmark). Immunoreactivity was detected using 3,3′‐diaminobenzidine (DAB), and the sections were counterstained with hematoxylin.

**TABLE 2:** Antibodies used for immunohistochemistry

Antigen	Type	Dilution	Antigen retrieval	Catalog No., RRID	Source
Astrocyte					
AQP4	Rabbit polyclonal	1:500	N.D.	Cat#: sc‐20812, RRID: AB_2274338	Santa Cruz Biotechnology, Dallas, TX, USA
GFAP	Rabbit polyclonal	Ready to use	N.D.	Cat#: IR524	DakoCytomation, Glostrup, Denmark
Cx43	Rabbit polyclonal	1:1000	N.D.	Cat#: ab11370, RRID: AB_297976	Abcam, Cambridge, UK
Cx43	Mouse monoclonal	1:100	N.D.	Cat#: 13‐8300, RRID: AB_86558	Thermo Fisher Scientific, Waltham, MA, USA
Cx30	Rabbit polyclonal	1:100	Autoclave/10 mM citrate buffer	Cat#: HPA014846, RRID: AB_1847135	Sigma–Aldrich, St Louis, MO, USA
Oligodendrocyte/myelin					
MAG	Rabbit polyclonal	1:400	N.D.	Cat#: HPA012499, RRID: AB_1854238	Sigma–Aldrich, St Louis, MO, USA
MOG	Rabbit polyclonal	1:1000	N.D.	Cat#: HPA021873, RRID: AB_1854055	Sigma–Aldrich, St Louis, MO, USA
MBP	Mouse monoclonal	1:200	N.D.	Cat#: sc‐271524, RRID: AB_10655672	Santa Cruz Biotechnology, Dallas, TX, USA
TPPP/p25α	Rabbit polyclonal	1:200	Autoclave/10 mM citrate buffer	Cat#: ab92305, RRID: AB_2050408	Abcam, Cambridge, UK
Cx32	Rabbit polyclonal	1:200	N.D.	Cat#: ab181374	Abcam, Cambridge, UK
Cx47	Rabbit polyclonal	1:500	Autoclave/10 mM citrate buffer	Cat#: SAB2100924, RRID: AB_10602971	Thermo Fisher Scientific, Waltham, MA, USA
OSP/Claudin‐11	Rabbit polyclonal	1:1000	N.D.	Cat#: ab53041, RRID: AB_2276205	Abcam, Cambridge, UK
NFASC	Rabbit polyclonal	1:200	N.D.	Cat#: HPA008832, RRID: AB_1854411	Sigma–Aldrich, St Louis, MO, USA
Caspr1	Mouse monoclonal	1:10000	Autoclave/10 mM citrate buffer	Cat#: ab252535	Abcam, Cambridge, UK
Macrophage/microglia					
CD68	Mouse monoclonal	1:200	Autoclave/10 mM citrate buffer	Cat#: M0814, RRID: AB_2314148	DakoCytomation, Glostrup, Denmark
Lymphocyte					
CD3	Mouse monoclonal	1:200	Autoclave/10 mM citrate buffer	Cat#: NCL‐L‐CD3‐PS1, RRID: AB_563544	Leica Biosystems, Nussloch, Germany
CD4	Rabbit monoclonal	1:200	Autoclave/10 mM citrate buffer	Cat#: M3350, RRID: AB_1660789	Spring Bioscience Corp, Pleasanton, CA, USA
CD8	Mouse monoclonal	1:200	Autoclave/10 mM citrate buffer	Cat#: H004	Nichirei Corp, Tokyo, Japan
CD20	Mouse monoclonal	Ready to use	Autoclave/10 mM citrate buffer	Cat#: IS604	DakoCytomation, Glostrup, Denmark
Neuron/axon					
p‐αSyn	Rabbit polyclonal	1:1000	N.D.	Cat#: ab51253, RRID: AB_869973	Abcam, Cambridge, UK
p‐αSyn	Mouse monoclonal	1:1000	N.D.	Cat#: 015‐25191, RRID: AB_2537218	Wako, Osaka, Japan

Abbreviations: AQP4, aquaporin‐4; Caspr1, contactin‐associated protein 1; Cat#, catalog number; CD, cluster of differentiation; Cx, connexin; GFAP, glial fibrillary acidic protein; MAG, myelin‐associated glycoprotein; MBP, myelin basic protein; MOG, myelin oligodendrocyte glycoprotein; N.D., not performed; NFASC, neurofascin; OSP, oligodendrocyte‐specific protein; p‐αSyn, phosphorylated alpha synuclein; RRID, Research Resource Identifier; TPPP/p25α, tubulin polymerization‐promoting protein.

### 2.3 Indirect immunofluorescence and confocal laser microscopy

Using the same set of paraffin sections described in Section 2.2, double immunofluorescence staining was performed with the following combinations of antibodies: rabbit polyclonal anti‐Cx32 and mouse monoclonal anti‐p‐αSyn; rabbit polyclonal anti‐claudin‐11/oligodendrocyte‐specific protein (OSP) and mouse monoclonal anti‐myelin basic protein (MBP); rabbit polyclonal anti‐neurofascin (NFASC) and mouse monoclonal anti‐MBP; rabbit polyclonal anti‐myelin oligodendrocyte glycoprotein (MOG) and mouse monoclonal anti‐contactin‐associated protein 1 (Caspr1); mouse monoclonal anti‐CD68 and rabbit monoclonal anti‐p‐αSyn; mouse monoclonal anti‐Cx43 and rabbit polyclonal anti‐Cx47; mouse monoclonal anti‐CD68 and rabbit polyclonal anti‐myelin‐associated glycoprotein (MAG). All sections were deparaffinized in xylene and rehydrated through a graded ethanol series. The sections were then incubated with primary antibodies at 4°C overnight. After rinsing, the sections were incubated with Alexa 488‐conjugated goat anti‐mouse IgG and Alexa 546‐conjugated goat anti‐rabbit IgG (Invitrogen, Waltham, MA, USA) and then counterstained with 4′,6‐diamidino‐2‐phenylindole. Images were captured using a confocal laser microscope system (Nikon A1, Nikon, Tokyo, Japan). We used a sequential multiple fluorescence scanning mode to avoid non‐specific overlap of colors and captured all images under the same magnification, laser intensity, gain and offset values, and pinhole setting conditions. To examine the specificity of immunohistochemical staining for Cxs, we first examined the specificity of primary antibodies against Cx30, Cx32, Cx43, and Cx47 in heart and liver tissue from a control case with progressive supranuclear palsy (as a negative control). No immunostaining was observed with anti‐Cx32 or ‐Cx30 antibodies in heart tissue or with anti‐Cx43 or ‐Cx47 antibodies in liver tissue, as previously reported [24, 25]. To test the cross‐reactivity of double immunofluorescence, we compared the double immunostaining patterns of anti‐Cx43 and ‐Cx47 antibodies with the single immunofluorescence patterns of anti‐Cx43 or ‐Cx47 antibodies in cerebellar white matter tissue from a control case. We also compared the double immunostaining patterns of anti‐Cx32 and ‐p‐αSyn antibodies with the single immunofluorescence patterns of anti‐Cx32 or ‐p‐αSyn antibodies in cerebellar white matter tissue with early‐stage demyelinating lesions from an MSA patient. The staining pattern of each antibody was similar between the single and double immunofluorescence staining, indicating no cross‐reactivity between anti‐Cx43 and ‐Cx47 antibodies or anti‐Cx32 and ‐p‐αSyn antibodies (Figure S1).

### 2.4 Pathological staging based on the degree of demyelination in MSA specimens

To assess the white matter pathology and demyelination in MSA, we selected the following anatomical sites: (i) transverse fibers of the pontine base and (ii) cerebellar white matter tracts receiving input signals from the spinocerebellar tracts, inferior olivary nucleus, and pontine nuclei called mossy and climbing fibers (Figure S1). We classified demyelinating lesions into three stages: (i) early demyelinating lesions characterized by a subtle reduction of myelin density (Stage I), (ii) intermediate demyelinating lesions characterized by a moderate reduction of myelin density (Stage II), and (iii) late demyelinating lesions characterized by a severe reduction of myelin density (Stage III) on the basis of KB staining, as previously reported [26].

### 2.5 Semi‐quantitative evaluation

We quantitatively measured the expression levels of glial Cxs, MAG, MOG, glial fibrillary acidic protein (GFAP), and CD68. The numbers of p‐αSyn‐positive cells, TPPP/p25α‐positive oligodendrocytes, claudin‐11/OSP‐, NFASC‐, and Caspr1‐positive paranodes, and immunocytes including T and B cells were counted manually. For the quantitative evaluation, sections immunohistochemically stained by each antibody were captured randomly by a light microscope (Olympus, Tokyo, Japan) at high magnification (400×). Manual counting and densitometric measurement were carried out in four regions of stained sections in each stage. For the densitometric measurements, four randomly selected regions of cerebellar afferent fibers were captured at 400× magnification using a light microscope (Olympus). Using Fiji ImageJ [27], the brown areas of DAB‐stained tissues were extracted and measured as areas of interest, which were expressed as % areas. Densitometric measurements were used for MAG, MOG, Cx43, Cx47, and GFAP immunostaining, as previously reported [28]. For the measurement of TPPP and Cx32 in myelin, four randomly selected regions of cerebellar afferent fibers were captured at 400× magnification, and myelin sheaths (but not oligodendrocyte somata) were manually extracted. Thereafter, DAB‐stained areas were measured as areas of interest, and TPPP‐ and Cx32‐stained areas in the myelin were calculated as % areas in myelin. In the double immunofluorescence evaluation of anti‐Cx32 and ‐p‐αSyn antibodies, the numbers of cells with co‐localization were manually counted at four random locations with a field of view of 400× magnification, and the co‐localization ratio was calculated. To evaluate Cx43/Cx47 GJ numbers by double immunofluorescence with anti‐Cx47 and ‐Cx43 antibodies, four regions of cerebellar white matter were first randomly captured at 400× magnification using confocal laser microscopy (Nikon). Heterotypic GJs consisting of Cx43 and Cx47, which were identified as either merged or adjacent to one another, were then manually counted.

### 2.6 Statistical analysis

Multi‐group comparisons with one‐way analysis of variance and Dunnett's post hoc test were conducted in this study because the distributions of values in each test followed a Gaussian distribution. Statistical analysis was performed using GraphPad Prism 8 (GraphPad Software, San Diego, CA, USA). A *p*‐value <0.05 was considered statistically significant.

### 2.7 Ethics statement

The study was approved by the Kyushu University Institutional Review Board for Clinical Research (22006‐00).

## 3 RESULTS

### 3.1 Distal oligodendrogliopathy (DO) is characteristic of demyelinating lesions in MSA

**FIGURE 1:**
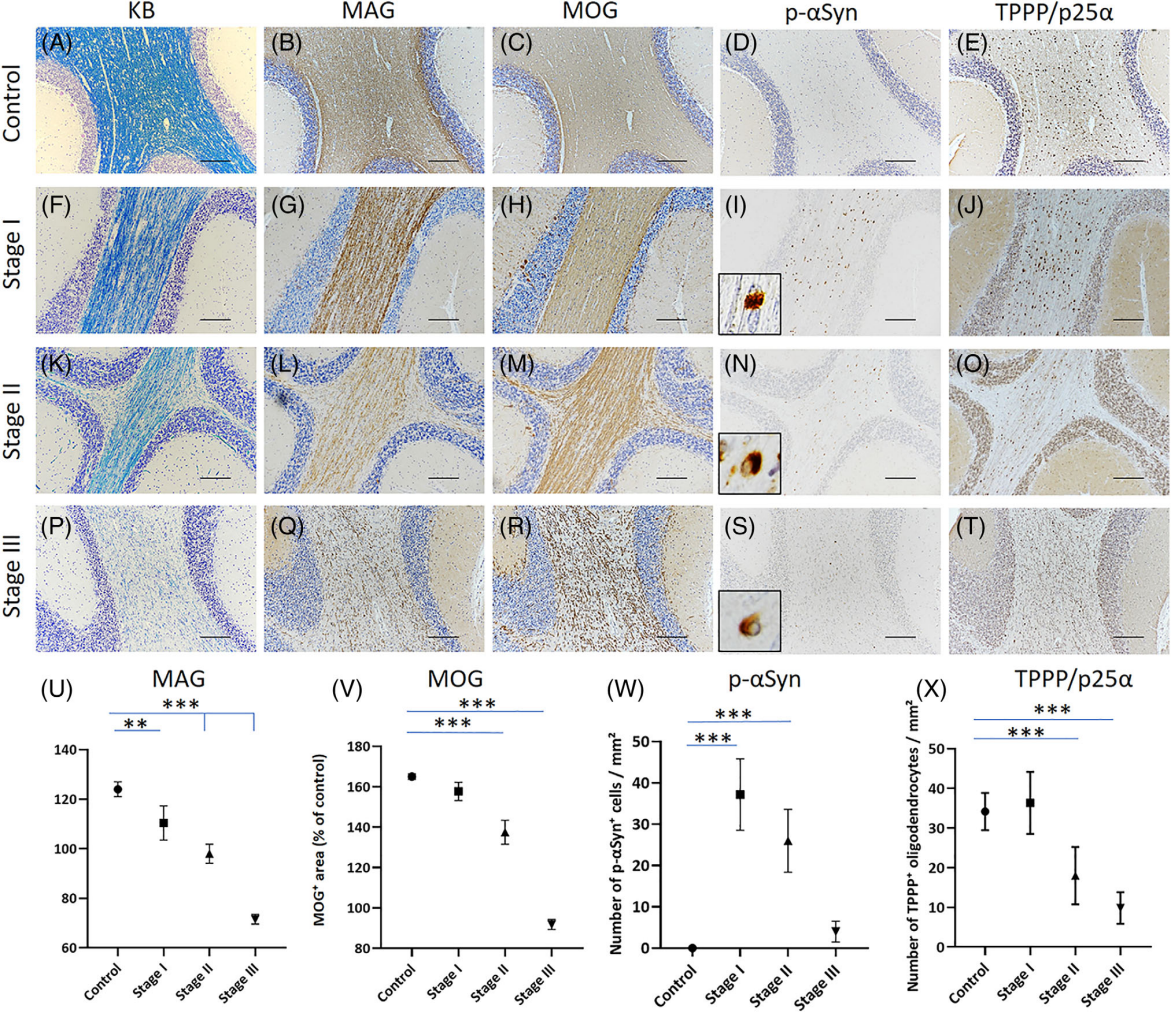
Myelin/oligodendrocyte protein loss in relation to phosphorylated α‐synuclein (p‐αSyn) accumulation. Representative images of a control specimen (myotonic dystrophy) (A–E), and multiple system atrophy (MSA) specimens of Stage I (MSA‐2) (F–J), II (MSA‐3) (K–O), and III disease (MSA‐1) (P–T) are shown with Klüver–Barrera (KB) (A, F, K, P), myelin‐associated glycoprotein (MAG) (B, G, L, Q), myelin oligodendrocyte glycoprotein (MOG) (C, H, M, R), p‐αSyn (D, I, N, S) with high‐magnification views (*insets*), and tubulin polymerization‐promoting protein (TPPP) (E, J, O, T) immunostaining. Graphs show MAG‐ (U) and MOG‐positive areas (% of control) (V) and the numbers of p‐αSyn‐positive cells (W) and TPPP‐positive oligodendrocytes (X) in each stage. Demyelination was not observed in the control specimen (A–C), and p‐αSyn‐positive cells were not observed (D), but TPPP/p25α‐positive oligodendrocytes were abundantly present (E) in the cerebellar white matter. The myelin density, determined by KB staining, gradually decreased from Stages I to III (F, K, P). The MAG expression level was already significantly reduced in Stage I and further decreased with advancing stage (G, L, Q, U). In contrast, the MOG expression level started to decrease in Stage II and further decreased in Stage III (H, M, R, V). In MSA, cytoplasmic accumulation of p‐αSyn in the oligodendrocytes was most frequently visible in Stage I, less frequently observed in Stage II, and observed in small numbers in Stage III (I, N, S, W, *inset*). The number of TPPP/p25α‐positive oligodendrocytes in the cerebellar white matter in Stage I was similar to that of the control and successively decreased in Stages II and III (J, O, T, X). Graphs display the mean ± SEM. *p < 0.05; **p < 0.01; ***p < 0.001. Scale bars: 200 μm

In MSA, reduced myelin density was visible in cerebellar afferent but not efferent fibers by KB staining (Figure S2E–G). At higher magnification, neither clustered thin myelin sheaths nor MOG‐immunoreactive oligodendrocytes, which have been reported to be characteristic of early remyelination in MS [29, 30, 31], were observed (Figure S2F,H), suggesting that the reduced myelin density was mainly attributable to demyelination rather than remyelination. On the basis of the most frequently observed demyelination grades in each specimen, five specimens (MSA‐2, 6, 8, 12, and 14) were classified into Stage I, three specimens (MSA‐3, 5, and 11) were classified into Stage II, and the remaining seven specimens (MSA‐1, 4, 7, 9, 10, 13, and 15) were classified into Stage III (Table 1). The disease duration was significantly longer at Stage III than that at Stage I (*p* = 0.0072, Figure S3). Consistent with the definition of the stage classification, KB staining showed a successive reduction of myelin density in the cerebellar white matter from Stages I to III (Figure 1A,F,K,P). In Stage I demyelinating lesions, MAG expression, located at the innermost layer of the myelin sheath, was preferentially decreased (Figure 1B,G,U), whereas that of MOG, located at the outermost layer of the myelin sheath, was relatively preserved (Figure 1C,H,V). These observations are consistent with DO type demyelination. In Stage II, MAG expression was markedly decreased, while the decrease in MOG remained subtle (Figure 1L,M,U,V). Both MAG and MOG were severely decreased in Stage III (Figure 1Q,R,U,V). Cytoplasmic accumulation of p‐αSyn was most frequently seen in Stage I oligodendrocytes but was not observed in controls (control versus Stage I, *p* < 0.001) (Figure 1D,I, *inset*, W). However, p‐αSyn accumulation became less frequent in Stage II (Figure 1N, *inset*) and was rare in Stage III specimens (Figure 1S, *inset*, W). The number of TPPP/p25α‐positive oligodendrocytes in the lesions was similar to that of control specimens at Stage I (control versus Stage I, *p* = 0.8743), while it was significantly decreased in Stages II (control versus Stage II, *p* < 0.001) and III (control versus Stage III, *p* < 0.001) compared with that in control specimens (Figure 1E,J,O,T,X). CD68‐positive microglia/macrophages exhibited abundant infiltration in Stage I demyelinating lesions compared with those at Stages II and III (control versus Stage I, *p* < 0.001) (Figure S4). In the cerebellar white matter, P2RY12‐positive homeostatic microglia were more abundant than CD68‐ and TMEM119‐positive cells in the control cases, whereas they were present at similar levels to CD68‐ and TMEM119‐positive cells in MSA Stage I (Figure S5A–C,F). Furthermore, in both control and Stage specimens, CD68‐ and TMEM119‐positive cells were present at similar levels to one another; however, both of these cell types were more abundant in Stage I than in controls (Figure S5A,B,D,E). Double immunofluorescence for CD68 and P2RY12 revealed double immunopositivity of these markers in controls and MSA (Figure S5G–J,O–R, arrow), while some P2RY12‐positive, CD68‐negative cells were observed in controls (Figure S5J, arrowhead) and some P2RY12‐negative, CD68‐positive cells were seen in MSA (Figure S5R, arrowhead). Cells with double immunopositivity for CD68 and TMEM119 were seen in both controls and MSA (Figure S5K–N,S–V, arrow), and some CD68‐positive, TMEM119‐negative cells were observed in MSA (Figure S5V, arrowhead). CD68‐positive cells without P2RY12 or TMEM119 staining were considered peripheral‐derived macrophages. CD3‐positive T cells were scattered in the demyelinating lesions, whereas CD20‐positive B cells were not observed in any stages (Figure S4). Quantitatively, among CD3‐positive T cells, similar numbers of CD4‐ and CD8‐positive T cells had infiltrated the lesions (Figure S4). Many CD68‐positive activated microglia/macrophages were located close to p‐αSyn‐positive GCIs and myelin, displaying foamy morphology in Stage I and II demyelinating lesions (Figure S6). These pathological features were consistent in the demyelinating lesions of all MSA specimens.

### 3.2 Membranous Cx32 in oligodendrocytic somata and myelin is lost and re‐distributed to the oligodendrocytic cytoplasm in MSA

**FIGURE 2:**
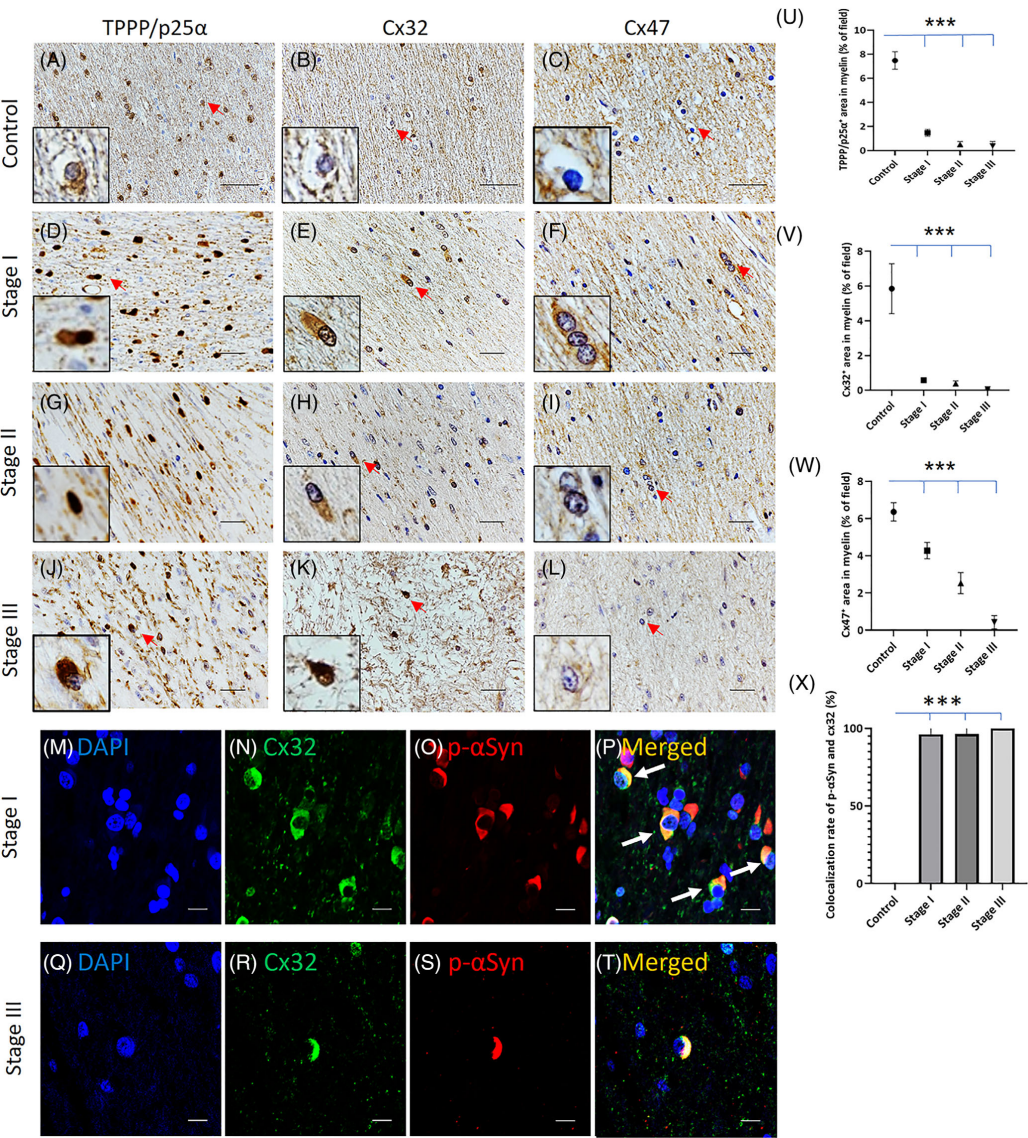
Early re‐distribution of the oligodendrocytic connexin (Cx) Cx32 from the membrane to the cytoplasm in multiple system atrophy (MSA). Representative images of a control specimen (myotonic dystrophy) (A–C) and MSA specimens with Stage I (MSA‐2) (D–F), II (MSA‐3) (G–I), and III disease (MSA‐1) (J–L) are illustrated by immunostaining with tubulin polymerization‐promoting protein (TPPP/p25α) (A, D, G, J), Cx32 (B, E, H, K), and Cx47 (C, F, I, L). Graphs show the percentage of TPPP/p25α‐ (U), Cx32‐ (V), and Cx47‐positive (W) areas in myelin fibers. TPPP/p25α, Cx32, and Cx47 were localized at the cellular membranes of oligodendrocytic somata and myelin in the cerebellar afferent fibers in the control specimen (A–C). In Stage I MSA, TPPP/p25α and Cx32 were extensively absent from the cellular membrane of oligodendrocyte somata and myelin and highly re‐distributed into the cytoplasm (D–E, U, V). Re‐distribution of these proteins was consistently seen in Stages II and III (G, H, J, K). Another oligodendrocytic Cx, Cx47, was also gradually decreased in a stage‐dependent manner but not re‐distributed into the cytoplasm in all stages (F, I, L, W). Double immunofluorescence detection of phosphorylated α‐synuclein (p‐αSyn) and oligodendrocytic Cx32 in Stage I cerebellar afferent fibers demonstrated that Cx32 was highly co‐localized with p‐αSyn‐positive glial cytoplasmic inclusions (GCIs) (M–P). Even in Stage III, the remaining p‐αSyn‐positive GCIs were co‐localized with Cx32 (Q–T). A graph shows the quantification of the co‐localization ratio of p‐αSyn and Cx32 (X). Scale bars: 25 μm (A–L), 10 μm (M–T). Graphs display the mean ± SEM. *p < 0.05; **p < 0.01; ***p < 0.001

In the control specimens, the expression of Cx32 and Cx47 in the cerebellar white matter was localized to the cellular membrane of oligodendrocytic somata and myelin, similar to that of TPPP/p25α (Figure 2A–C). In contrast, in demyelinating lesions in MSA, Cx32 and TPPP/p25α were nearly absent from the cellular membrane and myelin and were re‐distributed to the cytoplasm of oligodendrocytes even in Stage I (Figure 2D,E). In Stages II and III, Cx32 and TPPP/p25α were persistently re‐distributed in the oligodendrocytic cytoplasm, as seen in Stage I (Figure 2G,H,J,K). As a result, Cx32 and TPPP/p25α immunopositivity in myelin was markedly decreased in Stage I compared with that in control specimens (Figure 2U,V; control versus Stage I, *p* < 0.001). The expression of another oligodendrocytic Cx, Cx47, gradually decreased in a stage‐dependent manner; however, Cx47 was not re‐distributed to the cytoplasm in each stage (Figure 2F,I,L,W). Double immunofluorescence detection of p‐αSyn and oligodendrocytic Cx32 in demyelinating lesions revealed that Cx32 was highly co‐localized with p‐αSyn‐positive GCIs in all stages (Figure 2M–P,Q–T,X), whereas Cx47 was not co‐localized with p‐αSyn‐positive GCIs (data not shown), suggesting that Cx32 coaggregates with p‐αSyn in the oligodendrocytic cytoplasm beginning in the early stage of MSA.

### 3.3 Expression of paranodal proteins other than Cx32 is relatively preserved in early‐stage demyelinating lesions in MSA

**FIGURE 3:**
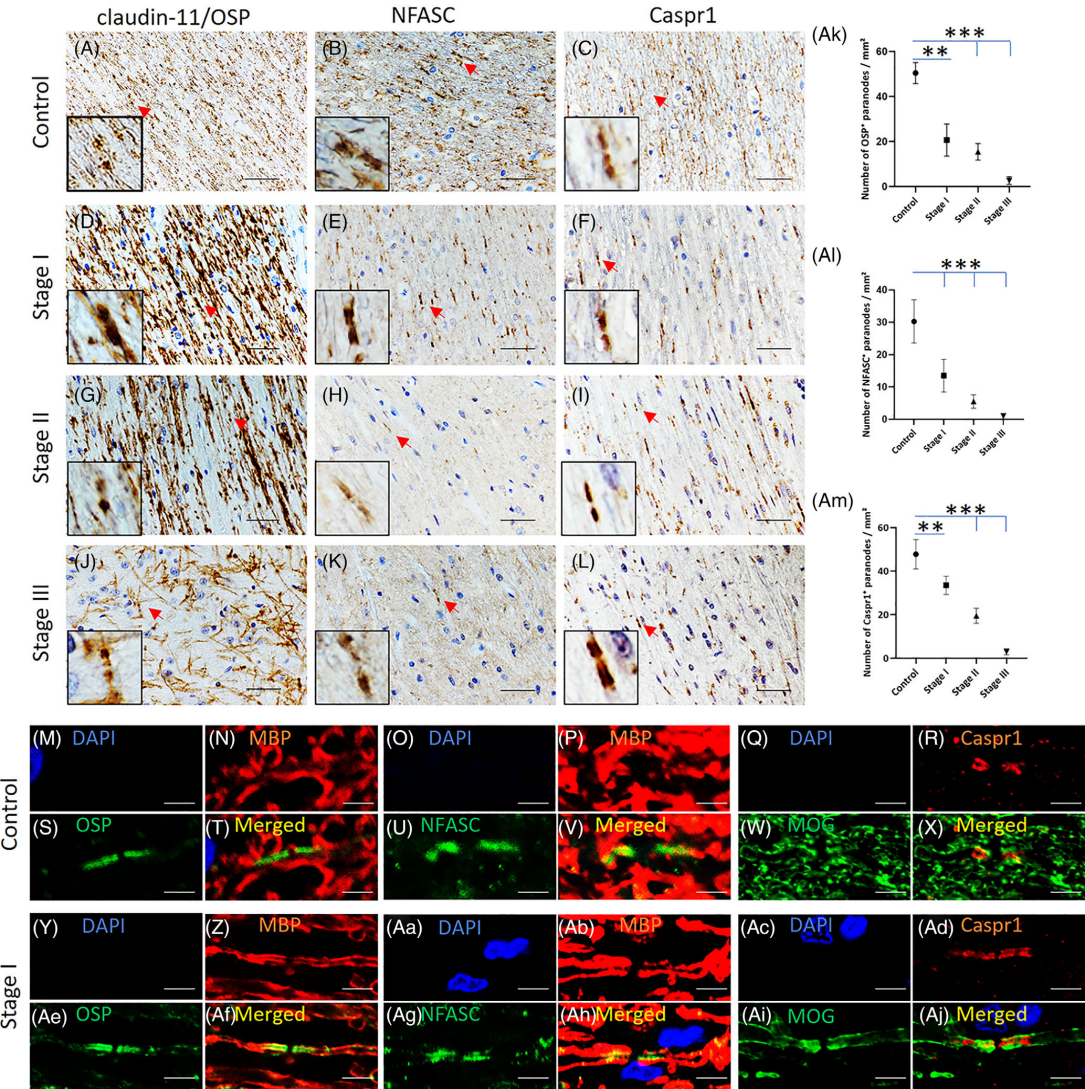
Expression pattern of claudin‐11/oligodendrocyte‐specific protein (OSP), neurofascin (NFASC), and contactin‐associated protein 1 (Caspr1) in demyelinated multiple system atrophy (MSA) lesions. Representative images of the cerebellar afferent fibers in a control specimen (limb‐girdle muscular dystrophy) (A–C) and MSA specimens with Stage I (MSA‐2) (D–F), II (MSA‐3) (G–I), and III disease (MSA‐1) (J–L) with OSP (A, D, G, I), NFASC (B, E, H, K), and Caspr1 (C, F, I, L) immunostaining are shown. Graphs show the numbers of OSP‐ (Ak), NFASC‐ (Al), and Caspr1‐positive paranodes (Am). In the control specimen, claudin‐11/OSP was present in the myelin sheath and paranodes (A, arrows, *inset*). Immunostaining for NFASC and Caspr1 showed a paranodal staining pattern (B, C, arrows, *inset*). In MSA, the expression levels of claudin‐11/OSP, NFASC, and Caspr1 gradually decreased with advancing stage (D–F, G–I, J–L, arrows, *inset*). Graphs show successive decreases in the numbers of paranodal protein‐positive paranodes in a stage‐dependent manner (Ak–Am). Double immunofluorescence labeling of claudin‐11/OSP and MBP, NFASC and MBP, and Caspr1 and myelin oligodendrocyte glycoprotein in a control specimen (M–X) and a Stage I MSA specimen (Y–Aj) shows preserved paranodal structures in both the control and MSA samples (M–Aj). Scale bars: 25 μm (A–L), 5 μm (M–Aj). Graphs display the mean ± SEM. **p < 0.01; ***p < 0.001

Next, we evaluated representative paranodal protein changes in demyelinating lesions in MSA because Cx32 is present at the paranodes [32]. The immunopositivity of claudin‐11/OSP, an intramyelinic and paranodal tight junction protein, NFASC, a paranode‐specific oligodendrocyte/axonal protein, and Caspr1, a paranode‐specific axonal protein, started to decrease at Stage I compared with that in control specimens (Figure 3A–F). Quantitatively, the numbers of claudin‐11/OSP‐, NFASC‐, and Caspr1‐positive paranodes were all significantly decreased, even at Stage I, and paranodal protein‐positive paranodes successively decreased in a stage‐dependent manner (Figure 3A–L, Ak–Am). In contrast, claudin‐11/OSP expression in compact myelin was relatively preserved, even at Stages II and III (Figure 3G,J). With double immunofluorescence labeling for claudin‐11/OSP and MBP, NFASC and MBP, and Caspr1 and MOG, claudin‐11/OSP, NFASC, and Caspr1 showed a similar paranodal staining pattern between MSA Stage I and controls (Figure 3M, Aj). This finding suggests that both paranodal protein expression and paranodal structures are relatively preserved in MSA (at least at Stage I), despite a pronounced decrease in paranodal and intramyelinic Cx32 (Figure 2E,R). Collectively, the loss of Cx32 seems to be an earlier pathological change than the decrease in other paranodal proteins during the process of MSA.

### 3.4 Marked down‐regulation of astrocytic Cx43 in early‐stage demyelinating lesions in MSA

**FIGURE 4:**
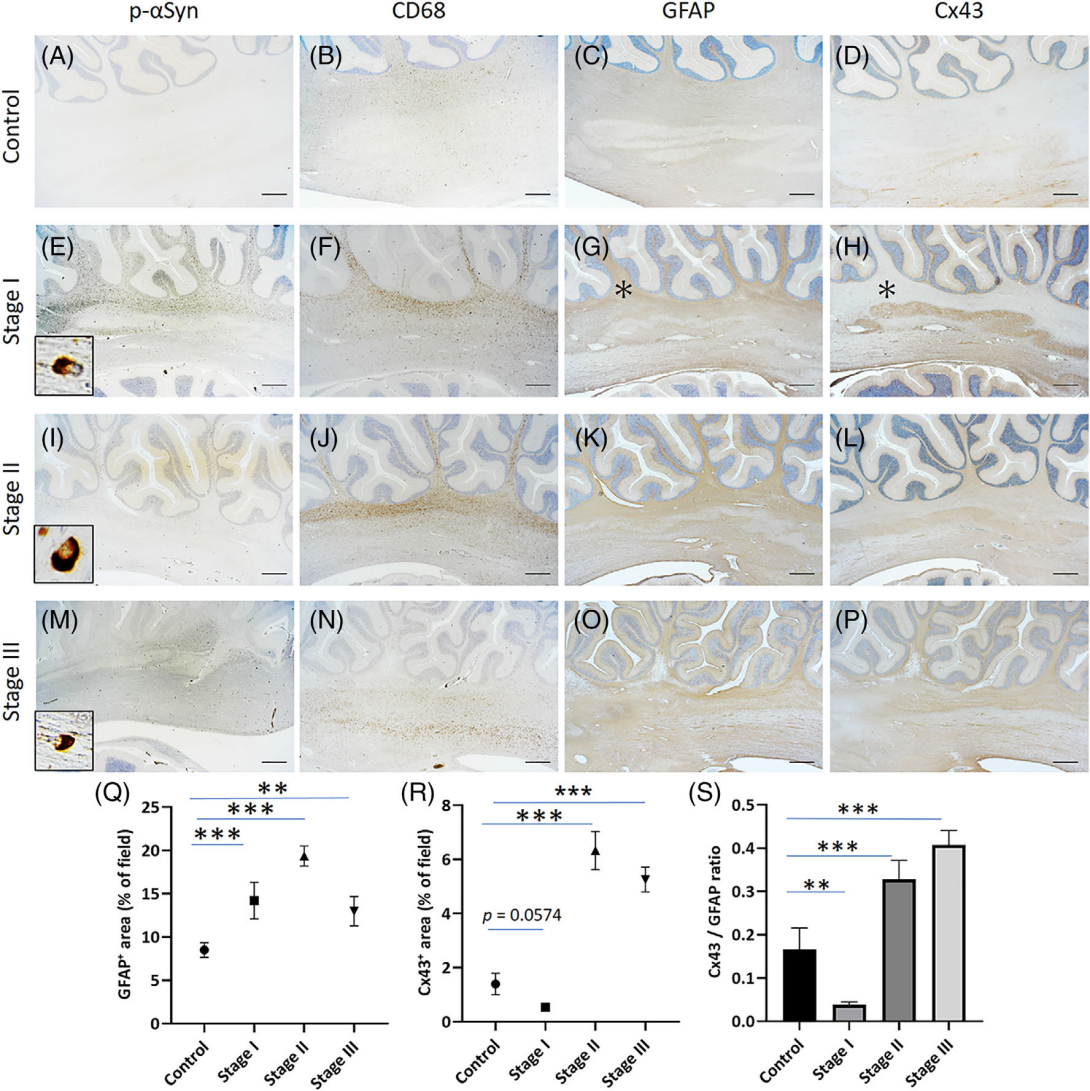
Stage‐dependent alterations of astrocytic connexin (Cx)43 in demyelinating lesions of multiple system atrophy (MSA). Representative images of the cerebellar afferent fibers in a control specimen (myotonic dystrophy) (A–D) and MSA specimens with Stage I (MSA‐2) (E–H), II (MSA‐3) (I–L), and III disease (MSA‐1) (M–P) are shown by phosphorylated α‐synuclein (p‐αSyn) (A, E, I, M) with high‐magnification views (*inset*), CD68 (B, F, J, N), glial fibrillary acidic protein (GFAP) (C, G, K, O), and Cx43 (D, H, L, P) immunostaining. Graphs show the percentage of GFAP‐ (Q) and Cx43‐positive areas (% of field) (R) and the ratios of areas stained positive for Cx43 and GFAP (GFAP/Cx43 ratio). In the control specimen, p‐αSyn‐immunopositive cells were undetectable (A). The degree of p‐αSyn accumulation was most prominent in Stage I (E), moderate in Stage II (I), and sparse in Stage III (M). Compared with scattered CD68‐positive microglia/macrophages in the control specimen (B), these cells were most abundant in Stage I (F), moderate in Stage II (J), and sparse in Stage III (N). Immunopositivity for GFAP (Q) was moderately up‐regulated in the cerebellar afferent fibers in Stage I (G, *asterisk*), highly up‐regulated in Stage II (K), and moderately up‐regulated in Stage III (O) compared with the control (C). The expression level of Cx43 was low in the cerebellar afferent fibers of the control specimen (D). In MSA, the expression of Cx43 was down‐regulated in demyelinating lesions of the cerebellar afferent fibers in Stage I despite the presence of GFAP‐positive astrocytes (G, H, *asterisk*). However, Cx43 was markedly up‐regulated at Stages II and III along with the activation of GFAP‐positive astrocytes (L, P, R). The ratio of Cx43 expression relative to GFAP‐positive reactive astrocytes (Cx43/GFAP ratio) was significantly decreased in Stage I but increased in Stages II and III (S). Scale bars: 1 mm (A–P). Graphs display the mean ± SEM. **p < 0.01; ***p < 0.001

The degrees of p‐αSyn accumulation and CD68‐positive microglia/macrophage infiltration in the cerebellar white matter were most prominent in Stage I compared with those in Stages II and III (Figure 1W, Figure 4A,B,E, *inset*, F, I, *inset*, J, M, *inset*, N, and Figure S4A,F,K,P,U). GFAP‐positive reactive astrocytes were increased in demyelinating lesions at all stages, reflecting astrogliosis, which was strongest at Stage II (control versus Stage I, *p* < 0.01; control versus Stage II, *p* < 0.001; control versus Stage III, *p* < 0.01) (Figure 4C,G,K,O,Q). In contrast, the immunoreactivity of Cx43 was decreased in Stage I demyelinating lesions (Figure 4H, *asterisk*) and increased in Stage II and III lesions (Figure 4L,P). Quantitatively, the Cx43 expression level showed a tendency to decrease in MSA at Stage I compared with that in controls (control versus Stage I, *p* = 0.0574) and was significantly increased in MSA at Stages II and III compared with that in control specimens (control versus Stage II, *p* < 0.001 and control versus Stage III, *p* < 0.001) (Figure 4R). However, compared with the control specimens, the expression ratio of Cx43‐ relative to GFAP‐positive astrocytes (Cx43/GFAP ratio) was significantly reduced in Stage I (control versus Stage I, *p* < 0.01) but markedly increased in Stage II and III specimens (control versus Stage II, *p* < 0.001 and control versus Stage III, *p* < 0.001) (Figure 4S). These changes were also consistently observed in the demyelinating lesions of pontine base transverse fibers (Figure S7). Expression of Cx30, another major astrocytic Cx, was predominantly observed in the dentate nucleus of the cerebellum and pontine nuclei. Immunoreactivity for Cx30 was not detected in reactive astrocytes in the affected white matter in any stage of MSA (Figure S8A,C,E,G). Aquaporin‐4 (AQP4), another astrocytic foot process protein, was abundantly expressed in the perivascular foot processes of astrocytes in the cerebellar white matter of control specimens (Figure S8B, *inset*). In MSA, AQP4 expression was slightly up‐regulated in Stage I demyelinating lesions but still preferentially showed a perivascular staining pattern; it was increased in Stage II and III lesions compared with control specimens (Figure S8B,D,F,H, *inset*). These findings indicate that astrocytic expression of Cx43 markedly fluctuated in demyelinating lesions in MSA according to the disease stage; it was specifically down‐regulated in the early stage and up‐regulated thereafter, even though reactive astrocytes were continuously present.

### 3.5 Disruption of Cx43/Cx47 GJs in demyelinating lesions through all stages of MSA

**FIGURE 5:**
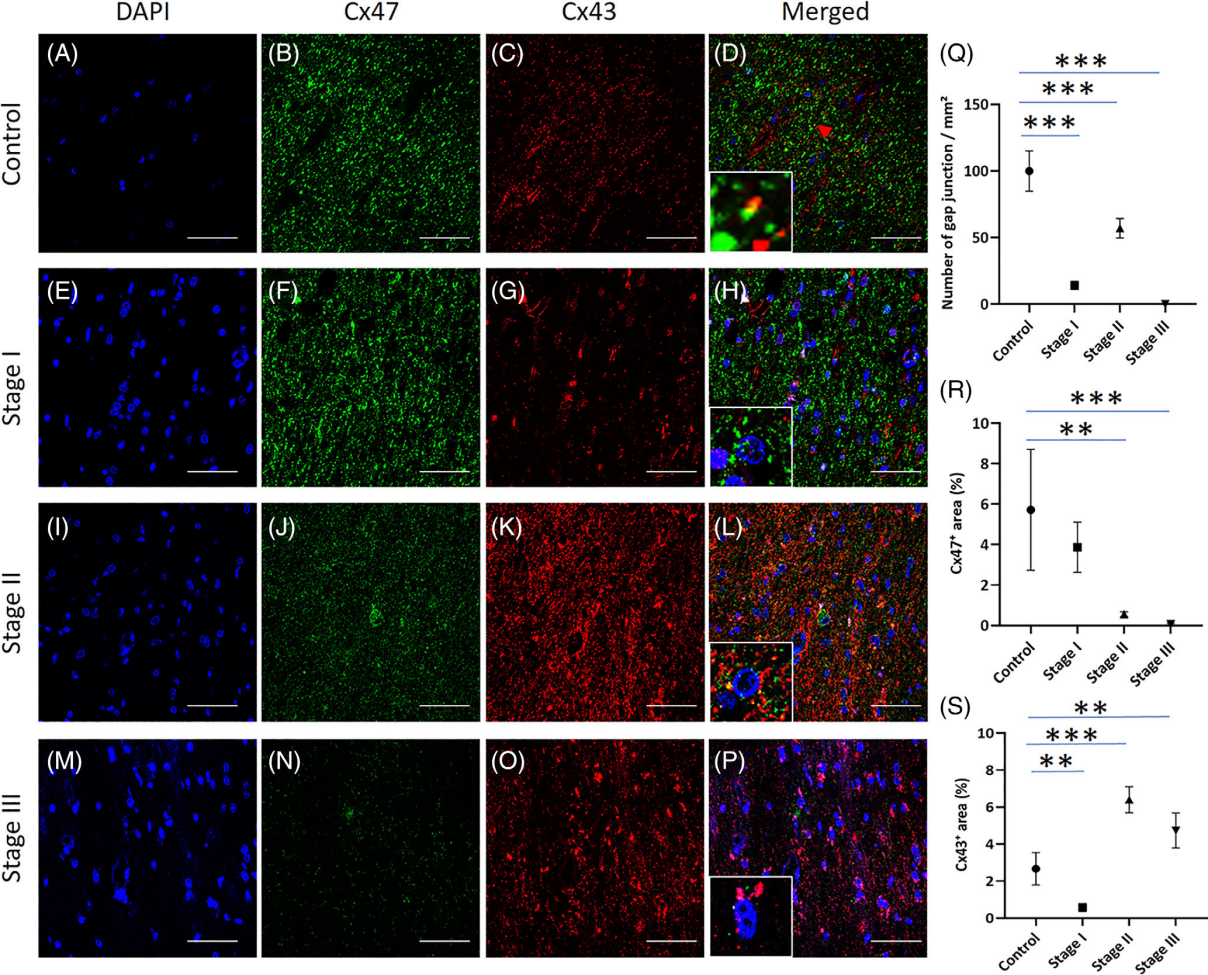
Disruption of connexin (Cx)43/Cx47 gap junctions (GJs) in the demyelinating lesions of multiple system atrophy (MSA). Representative images of the cerebellar afferent fibers in a control specimen (myotonic dystrophy) (A–D) and MSA specimens with Stage I (MSA‐2) (E–H), II (MSA‐3) (I–L), and III disease (MSA‐1) (M–P) are shown by double immunostaining with Cx47 and Cx43. Graphs show quantitation of Cx43/Cx47 GJs (Q), Cx43 (R), and Cx47 (S). Cx43 and Cx47 showed partial juxtaposition or co‐localization, suggestive of GJ plaque formation around oligodendrocytes in the control specimen (A–D, *inset*). In the cerebellar afferent fibers of Stage I MSA, Cx43 immunoreactivity was markedly decreased, whereas Cx47 immunoreactivity showed only subtle reduction, and Cx47 hemichannels were often detected (E–H, *inset*). In Stages II (I–L) and III (N–P), Cx47 immunoreactivity was decreased (J, N) while that of Cx43 was up‐regulated (K, O). In these stages, Cx43 hemichannels were often observed on astrocytes (L, P, *inset*) As a result, the number of Cx43/47 GJs was significantly decreased from Stages I to III (Q). Scale bars: 50 μm (A–P). Graphs display the mean ± SEM. **p < 0.01; ***p < 0.001

Finally, we evaluated heterotypic Cx43/Cx47 astrocyte/oligodendrocyte (A/O) GJs in cerebellar white matter lesions in MSA. Double immunofluorescence staining for Cx43 and Cx47 revealed bright, dot‐like Cx43 and Cx47 signals in astrocytes and around oligodendrocytes in control white matter specimens (Figure 5A–D). Double immunostaining for Cx43 and Cx47 showed partial juxtaposition or co‐localization suggestive of GJ plaque formation around oligodendrocytes in the controls (Figure 5D, *inset*). In Stage I demyelinating lesions in MSA, Cx43 immunoreactivity was markedly decreased in reactive astrocytes, while Cx47 immunoreactivity was relatively preserved in oligodendrocytes and myelin sheaths (Figure 5E–H). In contrast, Cx47 immunoreactivity was markedly decreased in Stage II and III lesions, while Cx43 immunoreactivity was markedly up‐regulated in Stage II and III lesions (Figure 5I–L,M–P,R,S). Quantitative analysis demonstrated that the number of Cx43/Cx47 GJs was significantly reduced in the demyelinating lesions from Stages I to III, although partial restoration of Cx43/Cx47 GJs was observed in Stage II lesions (Figure 5Q–S). These findings indicate that Cx43/Cx47 GJs are disrupted at the early stage as well as the intermediate to late stages of MSA because of down‐regulation of Cx43 and Cx47, respectively.

## 4 DISCUSSION

The present immunohistological study on MSA revealed the following glial Cx alterations: (1) Membranous expression of oligodendrocytic Cx32 was nearly absent in the early stage of MSA and never recovered in the following stages. (2) Cx32 was re‐distributed and co‐localized with p‐αSyn‐positive GCIs in the oligodendrocyte cytoplasm beginning in the early stage. (3) Oligodendrocytic Cx47 successively decreased in the membrane without re‐distribution into the cytoplasm as the stage progressed, while it was relatively preserved in the early stage compared with the absence of Cx32. (4) Cx43 was preferentially down‐regulated in activated astroglia in the early stage while it was markedly up‐regulated in the intermediate and late stages. (5) These glial Cx changes disrupted Cx43/Cx47 GJ channels, resulting in Cx47 hemichannels in the early stage and Cx43 hemichannels in the intermediate to late stages of MSA. In association with these glial Cx changes, we also found (6) DO type demyelination with massive infiltration of CD68‐positive microglia/macrophages and a considerable number of T cells but not B cells in early‐stage MSA lesions, and (7) nodal proteins, such as claudin‐11/OSP, NFASC, and Caspr1, gradually decreased with advancing stage. These findings are summarized in Table 3.

**TABLE 3:** Summary of characteristic changes in each pathological stage of demyelinating lesions in autopsied MSA patients

Control	GCI	TPPP/p25α		KB	MAG	MOG	Cx32		
	None	Oligodendrocytic membrane	Myelin	Myelin	Myelin	Myelin	Oligodendrocytic membrane	Myelin	Paranode
Stage I	↑↑↑	↓↓↓ (Cytoplasm)	↓↓↓	↓	↓	Preserved	↓↓↓ (Cytoplasm)	↓↓↓	↓↓↓
Stage II	↑↑	↓↓↓ (Cytoplasm)	↓↓↓	↓↓	↓↓	↓	↓↓↓ (Cytoplasm)	↓↓↓	↓↓↓
Stage III	↑	↓↓↓ (Cytoplasm)	↓↓↓	↓↓↓	↓↓↓	↓↓	↓↓↓ (Cytoplasm)	↓↓↓	↓↓↓

Control	NFASC	Caspr1	OSP		Cx47		GFAP	Cx43
	Paranode	Paranode	Myelin	Paranode	Oligodendrocytic membrane	Myelin	Astrocyte	Astrocyte foot process
Stage I	↓	↓	↓	↓	↓	↓	↑	↓
Stage II	↓↓	↓↓	↓↓	↓↓	↓↓	↓↓	↑↑↑	↑↑
Stage III	↓↓↓	↓↓↓	↓↓↓	↓↓↓	↓↓↓	↓↓↓	↑↑	↑↑

Abbreviations: Caspr1, contactin‐associated protein 1; Cx, connexin; GCI, glial cytoplasmic inclusion; GFAP, glial fibrillary acidic protein; KB, Klüver–Barrera; MAG, myelin‐associated glycoprotein; MOG, myelin oligodendrocyte glycoprotein; MSA, multiple system atrophy; NFASC, neurofascin; OSP, oligodendrocyte‐specific protein; TPPP/p25α, tubulin polymerization‐promoting protein; ↑, slightly increased; ↑↑, moderately increased; ↑↑↑, severely increased; ↓, slightly decreased; ↓↓, moderately decreased; ↓↓↓, severely decreased; (Cytoplasm), re‐distributed to oligodendrocyte cytoplasm.

Alterations of glial Cxs have been reported in a variety of neurological disorders affecting the CNS, such as MS [14], NMOSD [13], Alzheimer's disease [33, 34], Parkinson's disease [35], amyotrophic lateral sclerosis [36, 37], and epilepsy [38, 39]. In demyelinating diseases, oligodendrocytic Cx32 and Cx47 are markedly down‐regulated in acute lesions and persistently absent, even in chronic lesions, whereas astrocytic Cx43 is down‐regulated in activated astroglia in acute lesions but markedly up‐regulated in chronic lesions, reflecting astrogliosis [12, 13, 14]. These changes are in part consistent with the present findings for MSA.

It should be noted that for early lesions, MSA is unique in exhibiting widespread loss of oligodendrocytic Cx32 compared with the relative preservation of Cx47. Such early loss of Cx32 is striking, even compared with other nodal and myelin proteins. Intriguingly, Cx32 directly binds to αSyn/p‐αSyn in vivo as well as in vitro, resulting in preferential uptake of αSyn/p‐αSyn oligomers in oligodendrocytes [18, 40]. Co‐localization of Cx32 with p‐αSyn in GCIs in the oligodendrocyte cytoplasm in MSA was revealed by the present study. It is therefore speculated that Cx32, which is synthesized in the endoplasmic reticulum [41], coaggregates with p‐αSyn in GCIs. Because Cx32 has a rapid turnover with a half‐life of a few hours [41], Cx32 may not be properly delivered to myelin or paranodes because of coaggregation of Cx32 and p‐αSyn in the cytoplasm. This may in part explain the preferential loss of Cx32 in early lesions compared with Cx47, which does not coaggregate with p‐αSyn. Additionally, the persistent loss of Cx32 in the intermediate to late stages could result from more generalized dysfunctions and loss of oligodendrocytes.

Another striking feature in early‐stage lesions is extensive loss of astrocytic Cx43. We reported that the activation of microglia by interferon‐γ produced by Th1 cells causes astroglial Cx43 down‐regulation via secretion of interleukin‐1β, tumor necrosis factor‐α, and interleukin‐6 [19]. We and others have observed massive infiltration of activated microglia/macrophages and CD4^+^ T cells in early‐stage MSA lesions [3], which indicates that these inflammatory cells may contribute to astrocytic Cx43 loss, at least in early‐stage lesions. This is consistent with the recently highlighted crucial roles of peripheral immunocytes, particularly CD4^+^ T cells, in the pathophysiology of αSyn‐related disorders [3, 42].

In the CNS, Cx32 forms homotypic GJ channels between paranodal loops. The Cx32 GJ channels participate in potassium buffering upon axonal excitation at the node of Ranvier as well as lactate transfer to axons upon mitochondrial energy insufficiency [32]. Therefore, many mutations of *GJB1*, which encodes Cx32, cause X‐linked Charcot–Marie–Tooth disease [43], an inherited peripheral demyelinating neuropathy in which large CNS white matter lesions occasionally emerge [44, 45]. Furthermore, the *GJB1* p.P58S variant is responsible for X‐linked spinocerebellar ataxia, which presents with progressive ataxia and cerebellar atrophy with spinocerebellar and corticospinal tract demyelination [46]. Collectively, it is assumed that early and persistent loss of oligodendroglial Cx32 alone contributes to progressive demyelination and myelinated fiber loss in MSA through paranodal dysfunction, leading to impaired oligodendroglio‐axonal interaction (Figure S9).

We observed preferential MAG loss in early‐stage MSA lesions, which is thought to be an early neuropathological sign of oligodendrocyte dysfunction, leading to DO type demyelination [47]. DO type demyelination with preferential MAG loss has been reported in various demyelinating conditions, such as acute MS, NMOSD, Baló's concentric sclerosis, and progressive multifocal leukoencephalopathy [13, 47, 48, 49]. In MSA, DO lesions exclusively coexist with diffuse loss of Cx32 and Cx43, suggesting a close correlation between the two pathologies. Interestingly, DO type demyelination also coexists with Cx32/Cx47 and Cx43 loss in MS and NMOSD and is related to rapid deterioration to death [12, 13, 14]. Thus, loss of oligodendrocytic Cx32 and astrocytic Cx43 may facilitate DO type demyelination (Figure S9).

Mice lacking Cx32, Cx47, Cx43, or Cx30 display only subtle glial phenotypes, probably because of functional redundancy and compensation among various glial Cxs [16, 17]. However, dKO of two major oligodendrocytic Cxs, Cx32, and Cx47, results in severe demyelination, massive apoptotic oligodendrocyte death, and early mortality in mice [16]. Moreover, Cx32/Cx43 dKO mice develop white matter vacuolation and progressive loss of astrocytes and present sensorimotor impairment, seizure activity, and early mortality [16]. These findings indicate that loss of multiple glial Cxs results in demyelination and oligodendrocyte loss. This is also the case for MSA, which involves Cx32 and Cx43 loss in the early stage and Cx32 and Cx47 loss in the intermediate to late stages (Figure S9).

Cx43 and Cx47 form A/O GJ channels, which exchange ions, second messengers, and energy sources among glial syncytia to maintain CNS homeostasis. According to our results, A/O GJ channels are disrupted from the early to late stages of MSA, forming Cx47 hemichannels because of Cx43 loss in early‐stage lesions and Cx43 hemichannels because of Cx47 loss in intermediate‐ and late‐stage lesions. Such hemichannels could be hazardous to other cells through secretion of toxic substances, such as glutamate [50, 51], or to the cells themselves because of potassium ion efflux and influx of calcium and sodium ions [52, 53, 54]. Indeed, in NMOSD, anti‐AQP4 antibodies induced rapid alterations of glial Cxs, leading to GJ dysfunction, which profoundly decreased myelin density in a mixed glial culture system [55]. Accordingly, persistent loss of A/O GJ channels in MSA may also contribute to myelin loss and axonal degeneration via impairment of metabolic homeostasis and autocrine and paracrine cytotoxicity (Figure S9).

We found that nodal/paranodal proteins, such as claudin‐11/OSP, NFASC, and Caspr1, were gradually decreased from the early to late stages in myelinated fibers; this decrease may cause whole nodal dysfunction in MSA. Therefore, glial Cx alterations may initially induce DO and paranodal dysfunction in the early stage and later induce whole nodopathy, all of which could contribute to demyelination and myelinated axon degeneration in MSA (Figure S9).

The present study has some limitations. It was difficult to obtain sufficient numbers of autopsied tissues from MSA patients because of the rarity of this disease. Therefore, we were able to perform only a limited number of histochemical and immunofluorescence labeling assays to assess the expression profiles of Cxs and glial markers. Nonetheless, we successfully showed significant alterations of glial Cxs in each stage. As the numbers of specimens examined in each stage were not large, the present results should be regarded as preliminary and verified by a future large‐scale study.

In summary, we conclude that early and persistent alterations of oligodendrocytic and astrocytic Cxs are characteristic of MSA, and may lead to DO type demyelination and nodal pathology. Therefore, we propose that therapeutic strategies designed to protect or restore glial Cx impairment are warranted.

## AUTHOR CONTRIBUTIONS

Yuji Nishimura conceptualized and designed the study. Katsuhisa Masaki and Junichi Kira supervised the study. Dai Matsuse, Hiroo Yamaguchi, Tatsunori Tanaka, Shotaro Hayashida, Mitsuru Watanabe, Takuya Matsushita, Ryo Yamasaki, Noriko Isobe contributed to the tasks of analysing and interpreting data. Shoko Sadashima, Naokazu Sasagasako and Toru Iwaki contributed to sample collection and data acquisition. Katsuhisa Masaki and Eriko Matsuo assisted with the immunohistochemical experiments. Yuji Nishimura and Junichi Kira wrote the manuscript. All authors had final approval of the submitted manuscript.

## CONFLICT OF INTEREST

N. Isobe received research funds from Sumitomo Pharma, Mitsubishi Tanabe Pharma, Eisai, Nippon Boehringer Ingelheim, Japan Blood Products Organization, Chugai Pharmaceutical, Sumitomo Pharma, Mitsubishi Tanabe Pharma, Teijin Pharma, CSL Behring, Takeda Pharmaceutical, Novartis Pharma, and Biogen Japan and speaker honoraria from Novartis Pharma, Alexion, Takeda Pharmaceutical, Biogen Japan, Chugai Pharmaceutical, Mitsubishi Tanabe Pharma, FP Pharmaceutical, EA Pharma, Daiichi Sankyo, Eisai, Kyowa Kirin, CSL Behring, Otsuka Pharmaceutical, Sanofi, Teijin Healthcare, and Alnylam. J. Kira received research funds from Dainippon Sumitomo Pharma, Daiichi Sankyo, Mitsubishi Tanabe Pharma, and Kyowa Kensetsukougyo and consultancy and speaking fees and/or honoraria from Novartis Pharma, Mitsubishi Tanabe Pharma, CSL Behring, Biogen Japan, Teijin Health Care, the Takeda Pharmaceutical Company, Kyowa Kirin, Ono Pharmaceutical Co. Ltd., Alexion Pharmaceuticals Inc., Tsumura, Ricoh, EMC, and Eisai. The other authors declare that they have no conflict of interest.

## Supporting information


**Figure S1** Specificity of major primary antibodies used in this study. In heart tissue from a human progressive supranuclear palsy (PSP) patient, immunostaining for anti‐connexin (Cx)30 (A) and ‐Cx32 (B) was negative. In liver tissue from the PSP patient, immunostaining for anti‐Cx43 (C) and ‐Cx47 (D) was not detected. The single immunofluorescence staining for anti‐phosphorylated α‐synuclein (p‐αSyn) or anti‐Cx32 antibodies in cerebellar white matter tissue with Stage I demyelinating lesions of a multiple system atrophy (MSA) specimen (MSA‐2) (E–H) was similar to the double fluorescence staining of both antibodies (I–L). In controls, the single immunofluorescence staining for anti‐Cx43 and ‐Cx47 antibodies showed dot‐like staining patterns that were similar to the double immunostaining patterns of both antibodies (M–T). These observations suggest that there is no cross‐reactivity between anti‐p‐αSyn and ‐Cx32 antibodies or between anti‐Cx43 and ‐Cx47 antibodies. Scale bars: 25 μm (A–D), 10 μm (E–L), 20 μm (M–T).

**Figure S2** Overview of the analyzed regions in the cerebellum and pons. (A–D) Macroscopic view of the cerebellum and upper pons in a myotonic dystrophy specimen stained with hematoxylin and eosin (H&E) and Klüver–Barrera (KB). Cerebellar afferent fibers and transverse fibers of the pontine base were analyzed. (E–H) Multiple system atrophy (MSA) specimen with Stage I disease (MSA‐2). Demyelination was observed with KB staining in the cerebellar afferent (E, F) but not efferent (E, G) fibers. There were no thin myelin sheaths (F) or myelin oligodendrocyte glycoprotein (MOG)‐immunoreactive oligodendrocytes in MOG immunostaining (H). Scale bars: 1 cm (A–D), 1 mm (E), 25 μm (F–H).

**Figure S3** Disease duration according to demyelination stages in multiple system atrophy. The disease duration was significantly longer at Stage III than that at Stage I (*p* = 0.00011). The graph displays the mean ± SEM. yrs = years. ***p < 0.001.

**Figure S4** Inflammatory cell infiltration in the cerebellar afferent fibers in multiple system atrophy (MSA). Representative images of a control specimen (limb‐girdle muscular dystrophy) (A–E), and MSA specimens with Stage I (MSA‐2) (F–J), II (MSA‐3) (K–O), and III disease (MSA‐1) (P–T) with CD68 (A, F, K, P), CD3 (B, G, L, Q), CD4 (C, H, M, R), CD8 (D, I, N, S), and CD20 (E, J, O, T) immunostaining are shown. Graphs show the numbers of CD68‐ (U), CD3‐ (V), CD4‐ (W), CD8‐ (X), and CD20‐positive (Y) cells in each stage. In a control specimen, CD68‐positive resident microglia was visible in the cerebellar afferent fibers (A), and a few T cells were observed in blood vessels (B–E). In the afferent fibers in MSA, CD68‐positive microglia/macrophages exhibited the most abundant infiltration in Stage I compared with that in Stages II and III (F, K, P, U). The numbers of CD3‐, CD4‐, and CD8‐positive T cells were significantly increased in Stage I but not in Stages II and III (G–I, L–N, Q–S, V–X). CD20‐positive B cells were not observed in any stages (J, O, T, Y). Graphs display the mean ± SEM. *p < 0.05, ***p < 0.001. Scale bars: 50 μm (A–T).

**Figure S5** Expression patterns of CD68 in microglia and macrophages. Representative images of a control specimen (limb‐girdle muscular dystrophy) (A–C, G–N) and a multiple system atrophy (MSA) specimen with Stage I disease (MSA‐2) (D–F, O–V). CD68‐positive cells were as abundant as transmembrane protein 119 (TMEM119)‐positive cells, but were fewer than purinergic receptor P2RY12‐positive cells in both control (A–C) and MSA Stage I (D–F) specimens. Cells with double immunopositivity for CD68 and P2RY12 were observed in the control (G–J, arrow) and MSA (O–R, arrow) specimens, and a few P2RY12‐positive, CD68‐negative cells were also visible in both control and MSA specimens (G–J, arrowhead). Cells with double immunopositivity for CD68 and TMEM119 were observed in the control (K–N, arrow) and MSA (S–V, arrow) specimens, whereas some CD68‐positive, TMEM119‐negative cells were detected in MSA only (S–V, arrowhead). Scale bars: 200 μm (A–F), 50 μm (G–V).

**Figure S6** Activated microglia/macrophages surrounding phosphorylated α‐synuclein (p‐αSyn)‐positive glial cytoplasmic inclusions (GCIs). Representative images from a multiple system atrophy (MSA)‐2 specimen are shown (A–H). Double immunofluorescence staining for p‐αSyn and CD68 shows that CD68‐positive foamy microglia/macrophages surround p‐αSyn‐positive GCIs in the cerebellar afferent fibers in Stage I (A–D). However, such activated CD68‐positive microglia/macrophages do not phagocytose myelin‐associated glycoprotein‐positive myelin debris (E–H). Scale bars; 10 μm.

**Figure S7** Alterations of connexin (Cx)43 in pontine horizontal fibers in multiple system atrophy (MSA). Macroscopic images of the upper pons in Stage I in MSA (MSA‐2) are shown (A–C). Demyelination of the transverse fibers of the pontine base was subtle on Klüver–Barrera staining (A). Immunopositivity for phosphorylated α‐synuclein **(**p‐αSyn) and CD68 was preferentially detected in the demyelinated transverse fibers (B, C, arrowheads). Microscopic images of the horizontal fibers in a control specimen (myotonic dystrophy) (D–G) and MSA specimens with Stage I (MSA‐2) (H–K), II (MSA‐3) (L–O), and III disease (MSA‐4) (P–S) after immunostaining for p‐αSyn (D, H, L, P), CD68 (E, I, M, Q), glial fibrillary acidic protein (GFAP) (F, J, N, R), and Cx43 (G, K, O, S) are shown. Immunoreactivity for p‐αSyn, CD68, and GFAP was increased in the demyelinated transverse fibers in MSA compared with that in the control (D–F, H–J, L–N, P–R), as seen in the cerebellar afferent fibers (Figure 4). The Cx43 expression level was decreased in the demyelinated transverse fibers in Stage I but increased in Stages II and III (G, K, O, S). The red dotted lines indicate pontine base transverse fibers. In the control and Stage III specimens, the areas below the dotted line correspond to the transverse fibers. Scale bars: 1 cm (A–C), 25 μm (D–S).

**Figure S8** Expression pattern of connexin (Cx)30 and aquaporin‐4 (AQP4) in cerebellar afferent fibers with multiple system atrophy (MSA). Representative images of the cerebellar afferent fibers in a control specimen (myotonic dystrophy) (A, B) and MSA specimens with Stage I (MSA‐2) (C, D), II (MSA‐3) (E, F), and III disease (MSA‐1) (G, H) after immunostaining with Cx30 (A, C, E, G) and AQP4 (B, D, F, H) are shown. Cx30 expression was predominantly visible in the dentate nucleus and not observed in the cerebellar afferent fibers in both the control and MSA specimens (A, C, E, G). AQP4 was abundantly expressed in the perivascular foot processes of cerebellar white matter in the control specimen (B, *inset*). In the afferent fibers of MSA specimens, the AQP4 expression level was slightly up‐regulated in Stage I (D, *inset*) and obviously increased in Stages II (F, *inset*) and III (H, *inset*). Scale bars: 1 mm.

**Figure S9** Schematic drawing of glial connexin (Cx) changes leading to distal oligodendrogliopathy (DO) type demyelination and nodal/paranodal dysfunction in multiple system atrophy (MSA). The uppermost panel shows the normal patterns of glial Cxs, myelin proteins, and potassium buffering. In oligodendrocytes, Cx32 is present in the paranodal myelin and soma, while Cx47 is preferentially located in the soma. In astrocytes, Cx43 is expressed in foot processes. The black arrow indicates the potassium buffering flow via Cx gap junctions from axons to blood vessels and the pia mater. Blue arrows show the lactate transfer pathway from blood vessels through astrocytes, oligodendrocytes, and paranodal gap junctions to axons. The middle panel shows pathological changes in the early stage of demyelination in MSA (Stage I), where Cx32 is re‐localized to the oligodendrocytic cytoplasm. Paranodal proteins, such as claudin‐11/oligodendrocyte‐specific protein (OSP), contactin‐associated protein 1 (Caspr1), and neurofascin **(**NFASC), gradually decrease, but the subcellular localization is not altered. Furthermore, T cells and activated microglia prominently infiltrate demyelinating lesions in Stage I. Cx43 may be down‐regulated by cytokines (interleukin [IL]‐1β, tumor necrosis factor‐α, and IL‐6) secreted from microglia that are activated by interferon‐γ secreted from infiltrated T cells in Stage I (19). Cx47 may act as a hemichannel, causing efflux of cytotoxic glutamate and potassium as well as influx of calcium and sodium ions, which are harmful to oligodendrocytes. In early‐stage demyelinating lesions, loss of Cx32 and disruption of Cx43/47 gap junctions impairs potassium buffering and lactate translocation, exacerbating demyelination and axonal degeneration. The lowermost panel shows pathological changes in the late stage of demyelination in MSA patients (Stage III). The number of oligodendrocytes is markedly reduced, resulting in a marked decrease in Cx32 and Cx47. Expression of myelin‐associated glycoprotein, myelin oligodendrocyte glycoprotein, and paranodal proteins including NFASC, Caspr1, and Claudin‐11/OSP is markedly decreased. Expression of astrocytic Cx43 is up‐regulated along with astrogliosis and Cx43, which mainly forms hemichannels that secrete inflammatory cytokines and calcium.

## Data Availability

The data that support the findings of this study are available from the corresponding author upon reasonable request.
