# Supplementary figures and images for: Early and extensive alterations of glial connexins, distal oligodendrogliopathy type demyelination, and nodal/paranodal pathology are characteristic of multiple system atrophy
Source: Brain Pathol. 2022 Nov 11;33(3):e13131. doi: 10.1111/bpa.13131 (PMC10154368; doi:10.1111/bpa.13131)

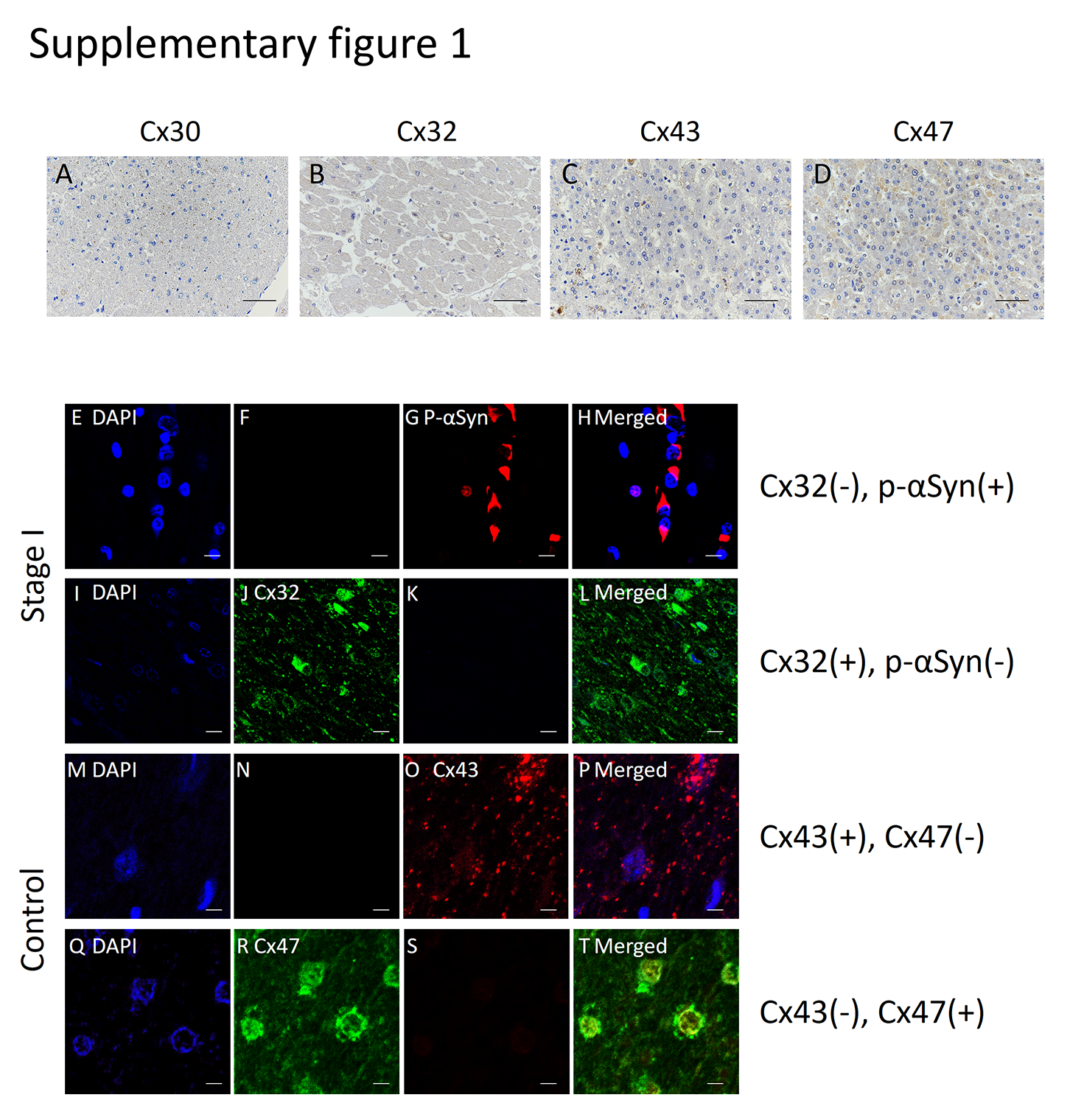

Supplement: Supplementary file 1 — Figure S1 Specificity of major primary antibodies used in this study. In heart tissue from a human progressive supranuclear palsy (PSP) patient, immunostaining for anti‐connexin (Cx)30 (A) and ‐Cx32 (B) was negative. In liver tissue from the PSP patient, immunostaining for anti‐Cx43 (C) and ‐Cx47 (D) was not detected. The single immunofluorescence staining for anti‐phosphorylated α‐synuclein (p‐αSyn) or anti‐Cx32 antibodies in cerebellar white matter tissue with Stage I demyelinating lesions of a multiple system atrophy (MSA) specimen (MSA‐2) (E–H) was similar to the double fluorescence staining of both antibodies (I–L). In controls, the single immunofluorescence staining for anti‐Cx43 and ‐Cx47 antibodies showed dot‐like staining patterns that were similar to the double immunostaining patterns of both antibodies (M–T). These observations suggest that there is no cross‐reactivity between anti‐p‐αSyn and ‐Cx32 antibodies or between anti‐Cx43 and ‐Cx47 antibodies. Scale bars: 25 μm (A–D), 10 μm (E–L), 20 μm (M–T). [file BPA-33-e13131-s006.tif]

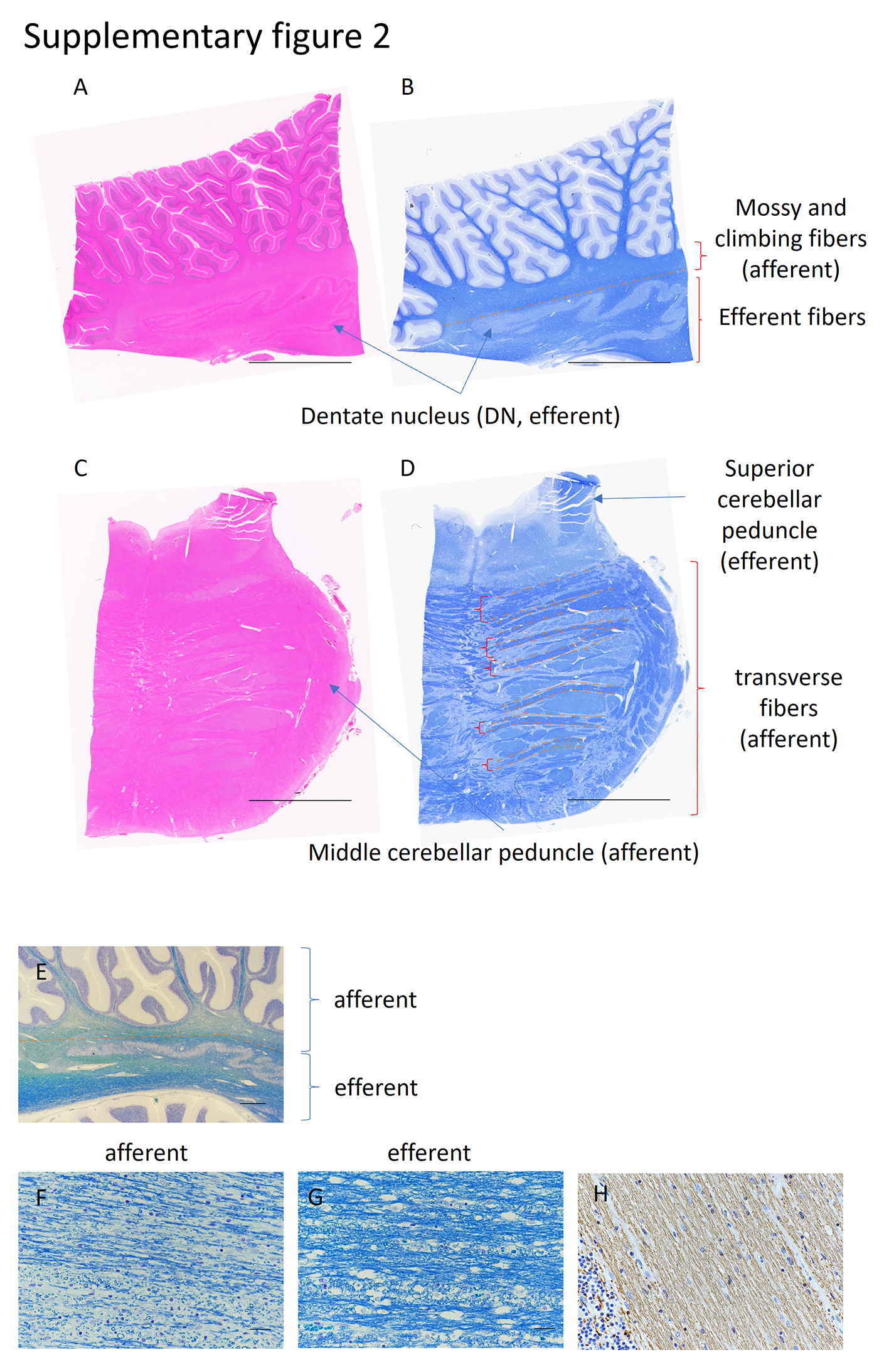

Supplement: Supplementary file 2 — Figure S2 Overview of the analyzed regions in the cerebellum and pons. (A–D) Macroscopic view of the cerebellum and upper pons in a myotonic dystrophy specimen stained with hematoxylin and eosin (H&E) and Klüver–Barrera (KB). Cerebellar afferent fibers and transverse fibers of the pontine base were analyzed. (E–H) Multiple system atrophy (MSA) specimen with Stage I disease (MSA‐2). Demyelination was observed with KB staining in the cerebellar afferent (E, F) but not efferent (E, G) fibers. There were no thin myelin sheaths (F) or myelin oligodendrocyte glycoprotein (MOG)‐immunoreactive oligodendrocytes in MOG immunostaining (H). Scale bars: 1 cm (A–D), 1 mm (E), 25 μm (F–H). [file BPA-33-e13131-s005.tif]

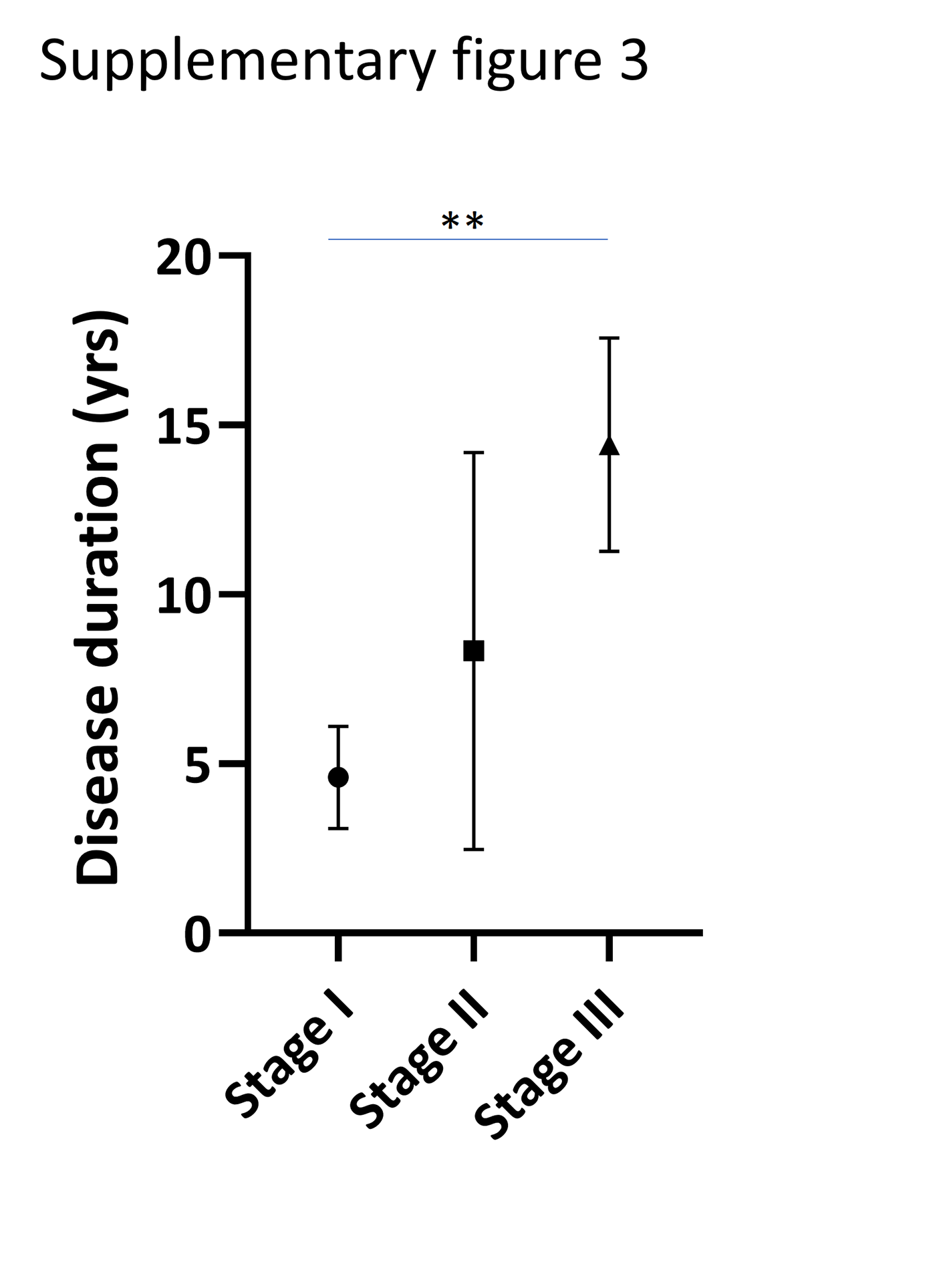

Supplement: Supplementary file 3 — Figure S3 Disease duration according to demyelination stages in multiple system atrophy. The disease duration was significantly longer at Stage III than that at Stage I (p = 0.00011). The graph displays the mean ± SEM. yrs = years. ***p < 0.001. [file BPA-33-e13131-s002.tif]

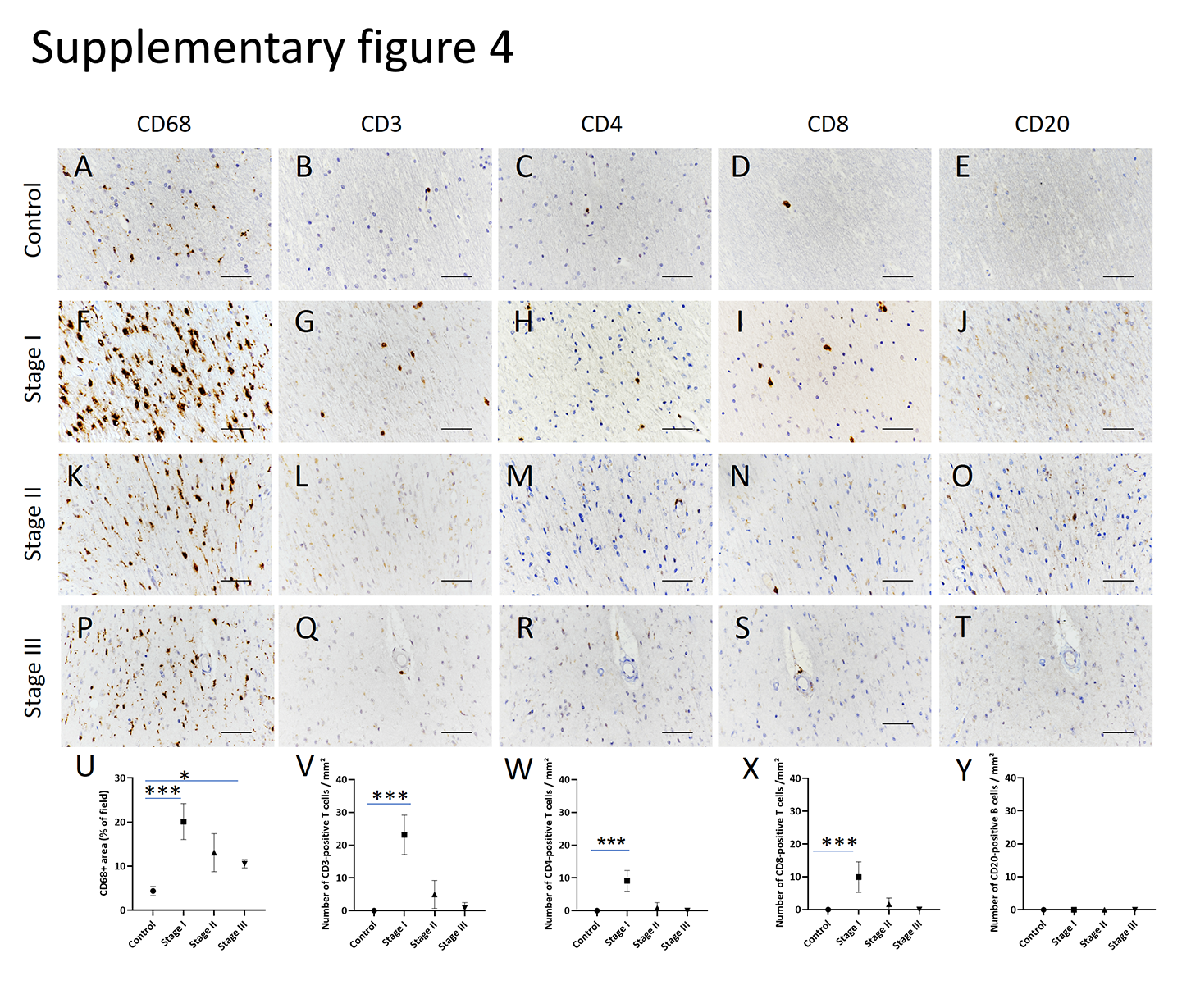

Supplement: Supplementary file 4 — Figure S4 Inflammatory cell infiltration in the cerebellar afferent fibers in multiple system atrophy (MSA). Representative images of a control specimen (limb‐girdle muscular dystrophy) (A–E), and MSA specimens with Stage I (MSA‐2) (F–J), II (MSA‐3) (K–O), and III disease (MSA‐1) (P–T) with CD68 (A, F, K, P), CD3 (B, G, L, Q), CD4 (C, H, M, R), CD8 (D, I, N, S), and CD20 (E, J, O, T) immunostaining are shown. Graphs show the numbers of CD68‐ (U), CD3‐ (V), CD4‐ (W), CD8‐ (X), and CD20‐positive (Y) cells in each stage. In a control specimen, CD68‐positive resident microglia was visible in the cerebellar afferent fibers (A), and a few T cells were observed in blood vessels (B–E). In the afferent fibers in MSA, CD68‐positive microglia/macrophages exhibited the most abundant infiltration in Stage I compared with that in Stages II and III (F, K, P, U). The numbers of CD3‐, CD4‐, and CD8‐positive T cells were significantly increased in Stage I but not in Stages II and III (G–I, L–N, Q–S, V–X). CD20‐positive B cells were not observed in any stages (J, O, T, Y). Graphs display the mean ± SEM. *p < 0.05, ***p < 0.001. Scale bars: 50 μm (A–T). [file BPA-33-e13131-s008.tif]

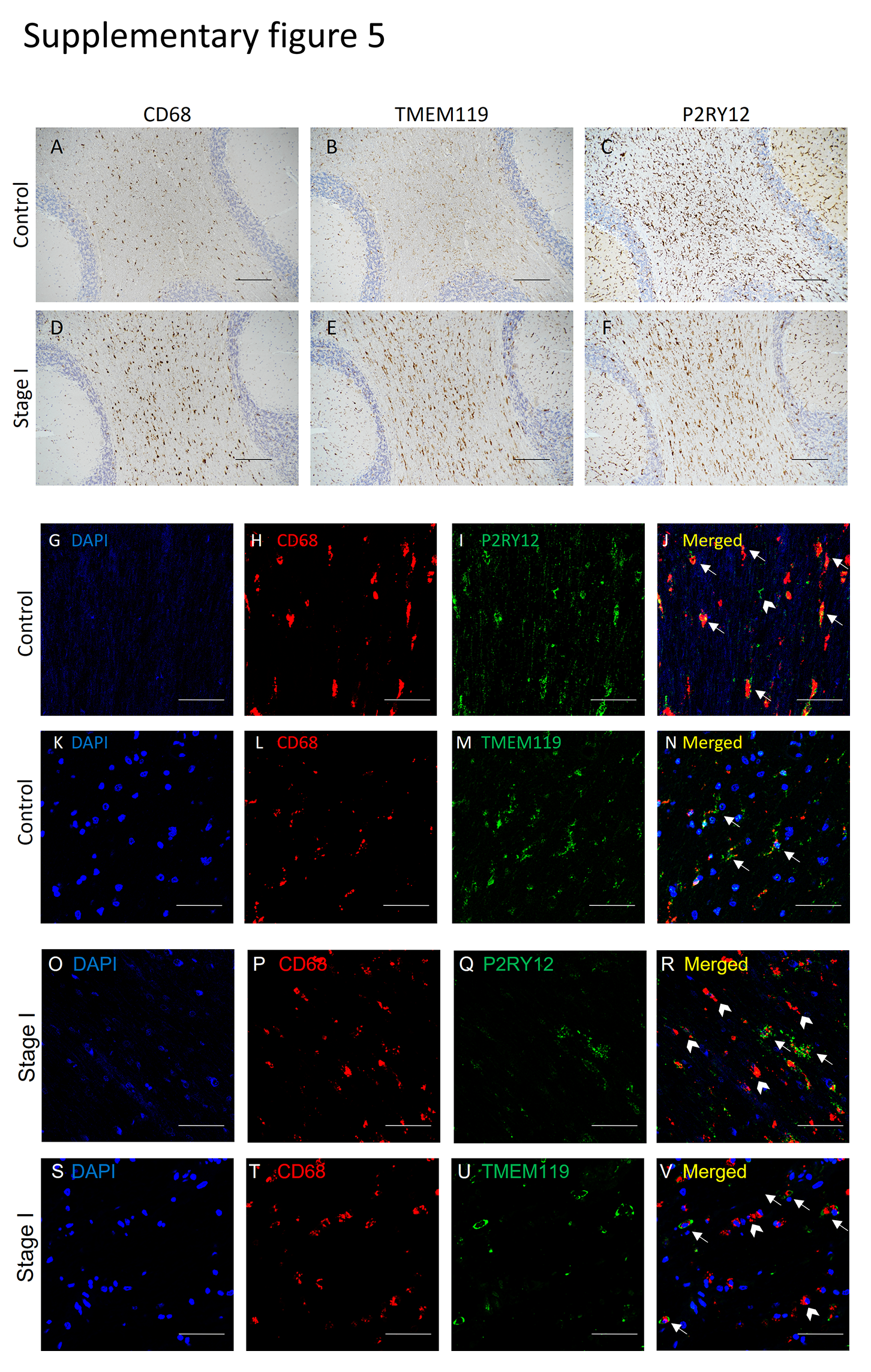

Supplement: Supplementary file 5 — Figure S5 Expression patterns of CD68 in microglia and macrophages. Representative images of a control specimen (limb‐girdle muscular dystrophy) (A–C, G–N) and a multiple system atrophy (MSA) specimen with Stage I disease (MSA‐2) (D–F, O–V). CD68‐positive cells were as abundant as transmembrane protein 119 (TMEM119)‐positive cells, but were fewer than purinergic receptor P2RY12‐positive cells in both control (A–C) and MSA Stage I (D–F) specimens. Cells with double immunopositivity for CD68 and P2RY12 were observed in the control (G–J, arrow) and MSA (O–R, arrow) specimens, and a few P2RY12‐positive, CD68‐negative cells were also visible in both control and MSA specimens (G–J, arrowhead). Cells with double immunopositivity for CD68 and TMEM119 were observed in the control (K–N, arrow) and MSA (S–V, arrow) specimens, whereas some CD68‐positive, TMEM119‐negative cells were detected in MSA only (S–V, arrowhead). Scale bars: 200 μm (A–F), 50 μm (G–V). [file BPA-33-e13131-s003.tif]

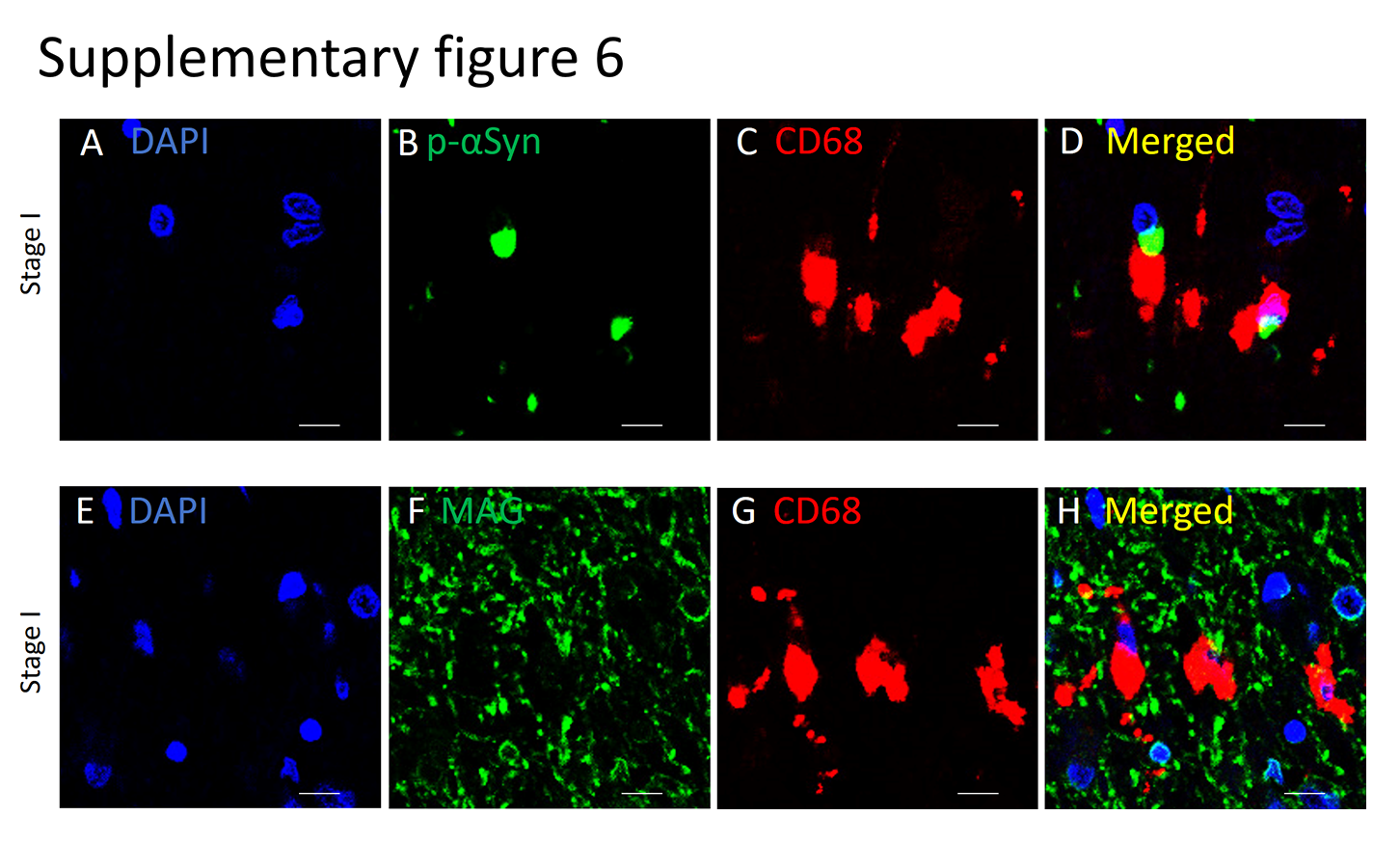

Supplement: Supplementary file 6 — Figure S6 Activated microglia/macrophages surrounding phosphorylated α‐synuclein (p‐αSyn)‐positive glial cytoplasmic inclusions (GCIs). Representative images from a multiple system atrophy (MSA)‐2 specimen are shown (A–H). Double immunofluorescence staining for p‐αSyn and CD68 shows that CD68‐positive foamy microglia/macrophages surround p‐αSyn‐positive GCIs in the cerebellar afferent fibers in Stage I (A–D). However, such activated CD68‐positive microglia/macrophages do not phagocytose myelin‐associated glycoprotein‐positive myelin debris (E–H). Scale bars; 10 μm. [file BPA-33-e13131-s009.tif]

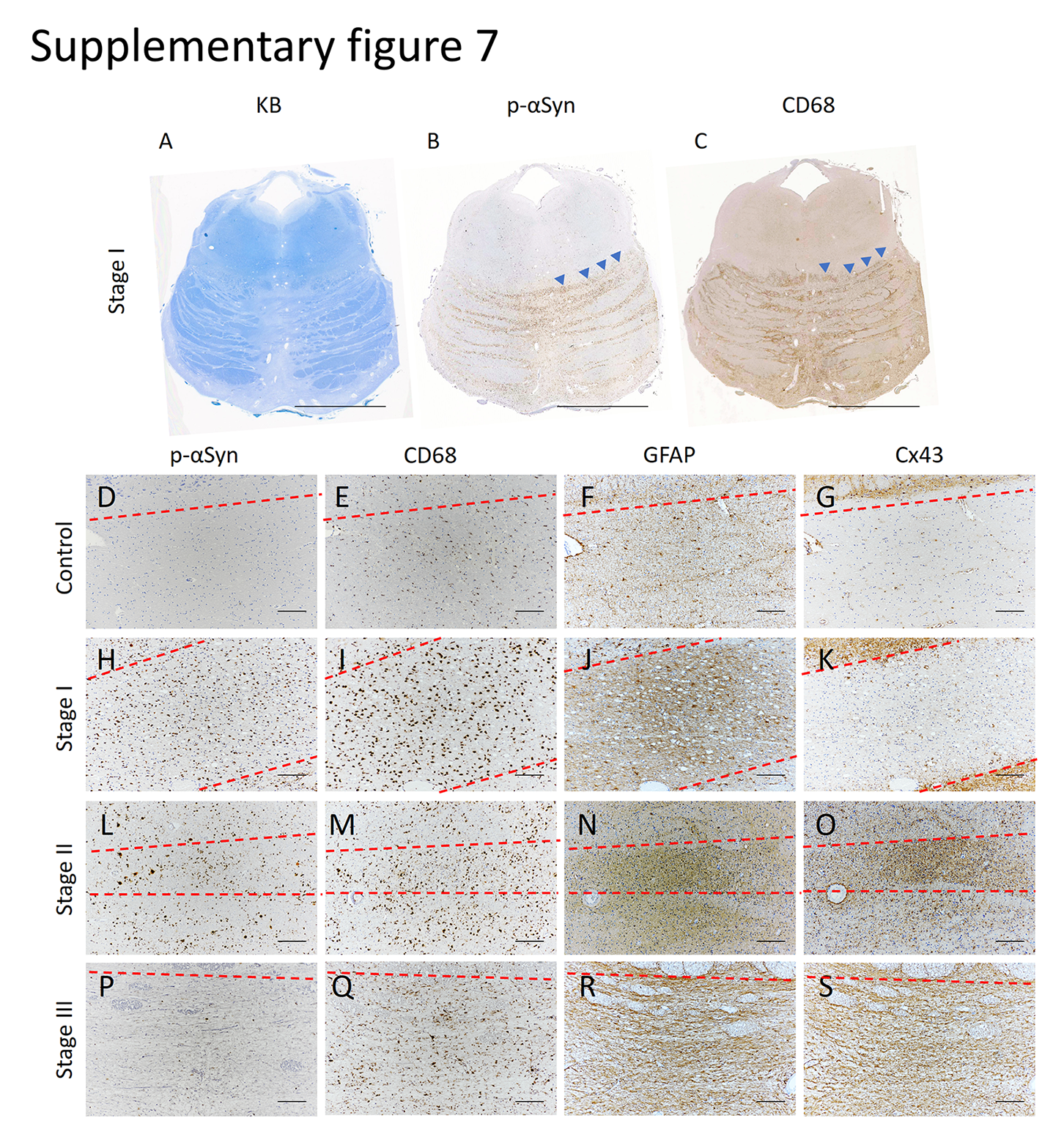

Supplement: Supplementary file 7 — Figure S7 Alterations of connexin (Cx)43 in pontine horizontal fibers in multiple system atrophy (MSA). Macroscopic images of the upper pons in Stage I in MSA (MSA‐2) are shown (A–C). Demyelination of the transverse fibers of the pontine base was subtle on Klüver–Barrera staining (A). Immunopositivity for phosphorylated α‐synuclein (p‐αSyn) and CD68 was preferentially detected in the demyelinated transverse fibers (B, C, arrowheads). Microscopic images of the horizontal fibers in a control specimen (myotonic dystrophy) (D–G) and MSA specimens with Stage I (MSA‐2) (H–K), II (MSA‐3) (L–O), and III disease (MSA‐4) (P–S) after immunostaining for p‐αSyn (D, H, L, P), CD68 (E, I, M, Q), glial fibrillary acidic protein (GFAP) (F, J, N, R), and Cx43 (G, K, O, S) are shown. Immunoreactivity for p‐αSyn, CD68, and GFAP was increased in the demyelinated transverse fibers in MSA compared with that in the control (D–F, H–J, L–N, P–R), as seen in the cerebellar afferent fibers (Figure 4). The Cx43 expression level was decreased in the demyelinated transverse fibers in Stage I but increased in Stages II and III (G, K, O, S). The red dotted lines indicate pontine base transverse fibers. In the control and Stage III specimens, the areas below the dotted line correspond to the transverse fibers. Scale bars: 1 cm (A–C), 25 μm (D–S). [file BPA-33-e13131-s007.tif]

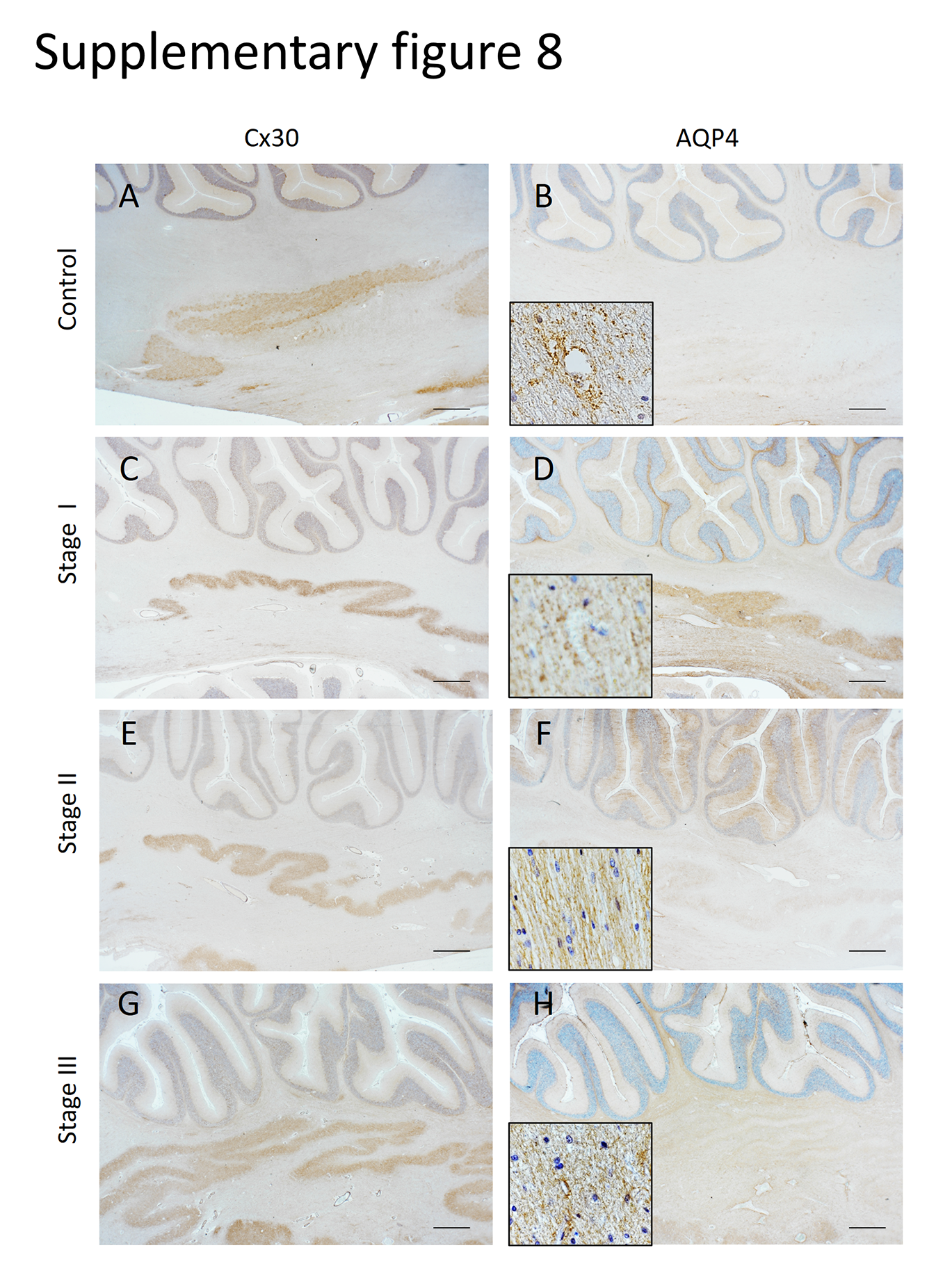

Supplement: Supplementary file 8 — Figure S8 Expression pattern of connexin (Cx)30 and aquaporin‐4 (AQP4) in cerebellar afferent fibers with multiple system atrophy (MSA). Representative images of the cerebellar afferent fibers in a control specimen (myotonic dystrophy) (A, B) and MSA specimens with Stage I (MSA‐2) (C, D), II (MSA‐3) (E, F), and III disease (MSA‐1) (G, H) after immunostaining with Cx30 (A, C, E, G) and AQP4 (B, D, F, H) are shown. Cx30 expression was predominantly visible in the dentate nucleus and not observed in the cerebellar afferent fibers in both the control and MSA specimens (A, C, E, G). AQP4 was abundantly expressed in the perivascular foot processes of cerebellar white matter in the control specimen (B, inset). In the afferent fibers of MSA specimens, the AQP4 expression level was slightly up‐regulated in Stage I (D, inset) and obviously increased in Stages II (F, inset) and III (H, inset). Scale bars: 1 mm. [file BPA-33-e13131-s001.tif]

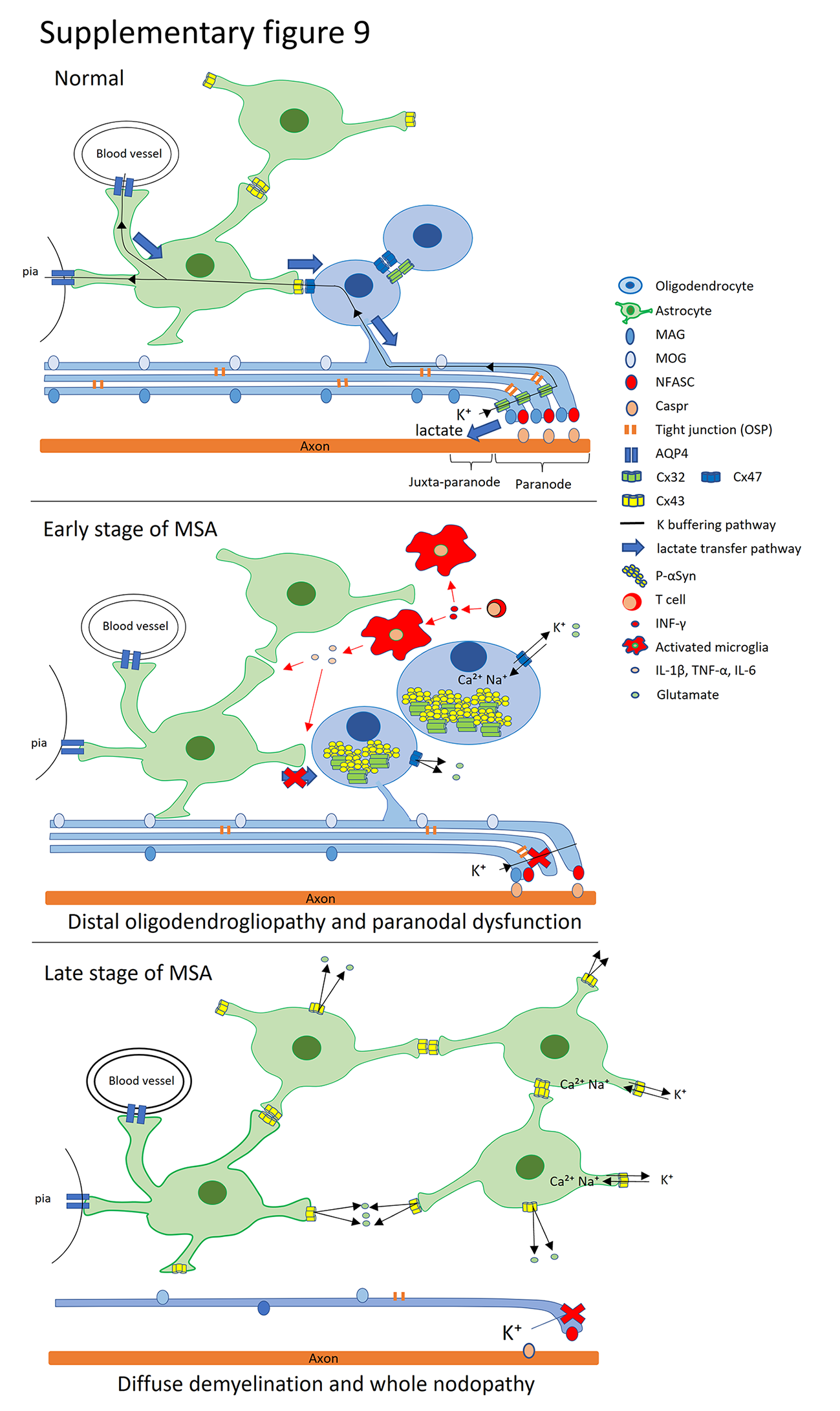

Supplement: Supplementary file 9 — Figure S9 Schematic drawing of glial connexin (Cx) changes leading to distal oligodendrogliopathy (DO) type demyelination and nodal/paranodal dysfunction in multiple system atrophy (MSA). The uppermost panel shows the normal patterns of glial Cxs, myelin proteins, and potassium buffering. In oligodendrocytes, Cx32 is present in the paranodal myelin and soma, while Cx47 is preferentially located in the soma. In astrocytes, Cx43 is expressed in foot processes. The black arrow indicates the potassium buffering flow via Cx gap junctions from axons to blood vessels and the pia mater. Blue arrows show the lactate transfer pathway from blood vessels through astrocytes, oligodendrocytes, and paranodal gap junctions to axons. The middle panel shows pathological changes in the early stage of demyelination in MSA (Stage I), where Cx32 is re‐localized to the oligodendrocytic cytoplasm. Paranodal proteins, such as claudin‐11/oligodendrocyte‐specific protein (OSP), contactin‐associated protein 1 (Caspr1), and neurofascin (NFASC), gradually decrease, but the subcellular localization is not altered. Furthermore, T cells and activated microglia prominently infiltrate demyelinating lesions in Stage I. Cx43 may be down‐regulated by cytokines (interleukin [IL]‐1β, tumor necrosis factor‐α, and IL‐6) secreted from microglia that are activated by interferon‐γ secreted from infiltrated T cells in Stage I (19). Cx47 may act as a hemichannel, causing efflux of cytotoxic glutamate and potassium as well as influx of calcium and sodium ions, which are harmful to oligodendrocytes. In early‐stage demyelinating lesions, loss of Cx32 and disruption of Cx43/47 gap junctions impairs potassium buffering and lactate translocation, exacerbating demyelination and axonal degeneration. The lowermost panel shows pathological changes in the late stage of demyelination in MSA patients (Stage III). The number of oligodendrocytes is markedly reduced, resulting in a marked decrease in Cx32 and Cx47. Expression o [file BPA-33-e13131-s004.tif]
